# The causal relationship between 233 metabolites and coronary atherosclerosis: a Mendelian randomization study

**DOI:** 10.3389/fcvm.2024.1439699

**Published:** 2024-12-12

**Authors:** Hongwei Zhang, Xiaoyu Zheng, Zian Yan, Lijun Guo, Yuan Zheng, Dawu Zhang, Xiaochang Ma

**Affiliations:** ^1^ National Clinical Research Center for Chinese Medicine Cardiology, Xiyuan Hospital, China Academy of Chinese Medical Sciences, Beijing, China; ^2^ State Key Laboratory of Traditional Chinese Medicine Syndrome, Xiyuan Hospital, China Academy of Chinese Medical Sciences, Beijing, China; ^3^ Institute of Basic Medical Sciences, Xiyuan Hospital of China Academy of Chinese Medical Sciences, Beijing, China; ^4^ Graduate School, Beijing University of Chinese Medicine, Beijing, China

**Keywords:** coronary atherosclerosis, metabolites, Mendelian randomization, causal relationship, two-sample

## Abstract

**Objective:**

To investigate the causal relationship between 233 newly reported metabolites and coronary atherosclerosis through Mendelian randomization analysis.

**Methods:**

Five different methods were used to perform Mendelian randomization analysis on the 233 metabolites and coronary atherosclerosis, with inverse variance weighting as the primary result, supplemented by other methods.

**Results:**

The analysis identified that certain metabolites increase the susceptibility risk of coronary atherosclerosis, including: Total fatty acids (OR = 1.40, 95% CI: 1.28–1.53, *P* < 0.001), Saturated fatty acids (OR = 1.44, 95% CI: 1.30–1.60, *P* < 0.001), Serum total triglyceride levels (OR = 1.33, 95% CI: 1.22–1.46, *P* < 0.001), Conjugated linoleic acid (OR = 1.16, 95% CI: 1.04–1.30, *P* = 0.007). Conversely, certain metabolites were found to reduce the occurrence of coronary atherosclerosis, such as: Cholesteryl esters to total lipids ratio in medium HDL (OR = 0.73, 95% CI: 0.67–0.78, *P* < 0.001), Cholesteryl esters to total lipids ratio in large HDL (OR = 0.64, 95% CI: 0.58–0.71, *P* < 0.001), Total cholesterol to total lipids ratio in medium HDL (OR = 0.71, 95% CI: 0.65–0.77, *P* < 0.001).

**Conclusion:**

There is a close relationship between metabolites and the occurrence of coronary atherosclerosis. This study conducted Mendelian randomization analysis on the causal relationship between 233 metabolites and coronary atherosclerosis, providing potential new insights for the treatment of this disease.

## Introduction

Coronary atherosclerosis, the most common form of arteriosclerosis, is a major cause of coronary heart disease. It leads to the narrowing or blockage of coronary arteries, resulting in myocardial ischemia, hypoxia, and, in severe cases, infarction and other cardiovascular diseases (1). Emerging research has increasingly focused on metabolites, such as lipids, lipoproteins, and fatty acids, in the progression of atherosclerosis, as these metabolites influence coronary atherosclerosis through various pathways. For example, indole-3-propionic acid (IPA), a gut microbiota-derived metabolite, has been shown to mitigate atherosclerosis by promoting cholesterol efflux, while deficiency in indoleamine 2,3-dioxygenase 1 (IDO1) exacerbates atherosclerosis progression by disrupting kynurenine metabolism and inducing osteogenic changes in vascular smooth muscle cells (2, 3). These findings underscore the inextricable link between coronary atherosclerosis and metabolites.

Given the significant role of these metabolites, it is crucial to focus on the relationship between metabolites and coronary atherosclerosis. Fortunately, a study by Karjalainen et al. (4) identified 233 circulating metabolic biomarkers, including 213 lipids, lipoprotein parameters, and fatty acids, across over 135,000 participants, offering a comprehensive profile to enhance our understanding of atherosclerosis.

Mendelian randomization (MR) is a research method that uses genetic variation as instrumental variables to evaluate causal effects (5). In MR analysis, genetic variants, typically single nucleotide polymorphisms (SNPs), serve as instrumental variables for particular risk factors. This method relies on Mendel's second law, where genetic alleles segregate independently during gamete formation, similar to the treatment assignment in randomized controlled trials (RCTs), thus reducing confounding (6). MR studies enable a deeper understanding of the causal relationship between metabolites and coronary atherosclerosis, offering new insights for disease prevention and potential therapeutic targets.

## Materials and methods

### Study design

**Figure 1:**
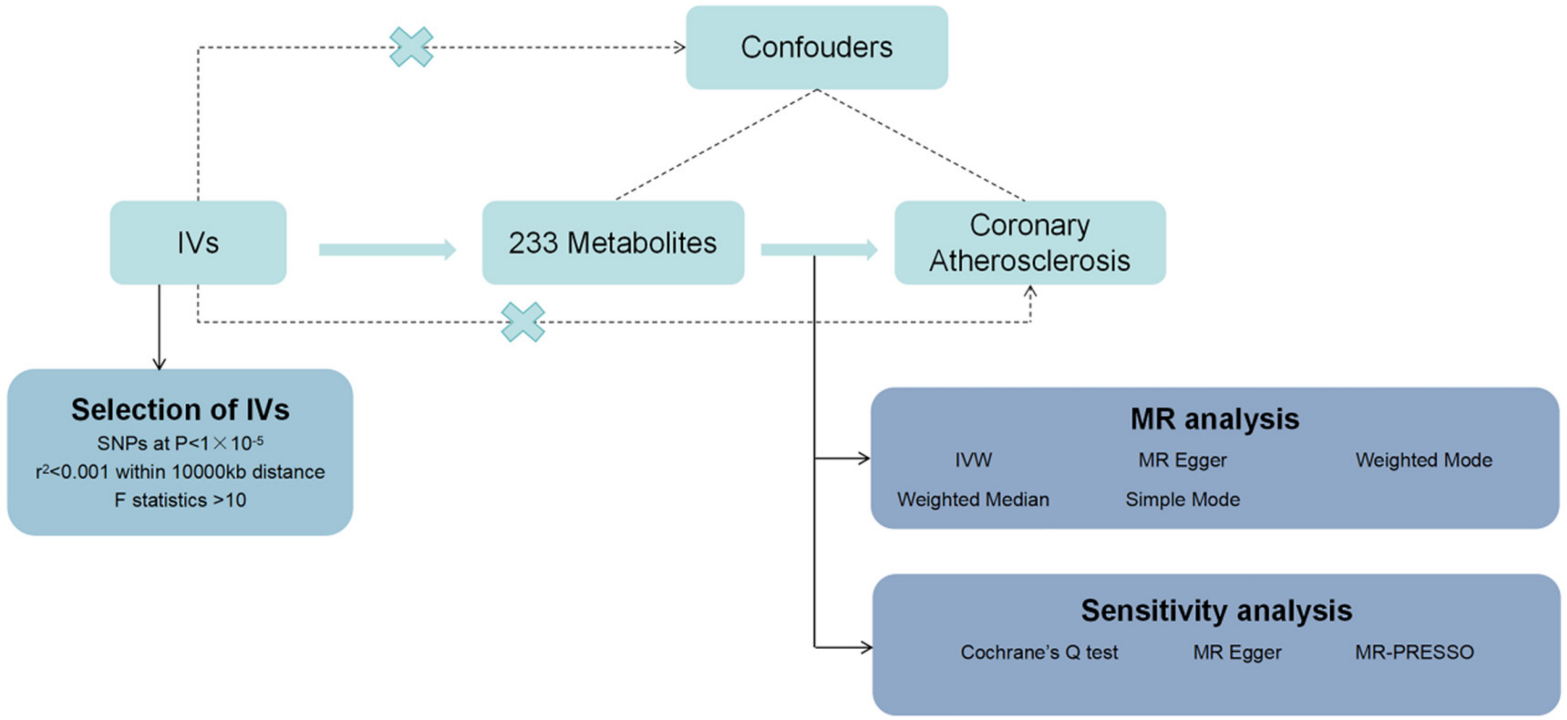
Flowchart of the design of this study.

In this study, we used MR analysis to investigate the causal relationship between 233 metabolites and coronary atherosclerosis. Methods such as heterogeneity tests and gene pleiotropy tests were applied to verify the stability of these causal relationships. The flowchart of the study is shown in Figure 1. The analysis was conducted under the three fundamental assumptions of MR:

1. Instrumental variables are strongly associated with the metabolites.
  The primary criterion for selecting instrumental variables is a significant association with the metabolites under study. If the association is weak, MR analysis may lack sufficient statistical power, potentially leading to biased or imprecise results. Thus, we prioritize genetic variants that are strongly correlated with the metabolites to ensure the validity and accuracy of causal inference.
2. The selected instrumental variables are independent of any potential confounding factors.
  This assumption ensures that the causal pathway remains singular, unaffected by confounding bias. Since genetic variants are determined at birth and are generally unrelated to environmental factors or other potential confounders in a randomly distributed population, MR is less prone to confounding issues compared to traditional observational studies.
3. Instrumental variables influence the risk of coronary atherosclerosis solely through the metabolites, without any other pathways.
  This assumption guarantees that the instrumental variables impact the outcome exclusively through their association with the metabolites, avoiding other direct or indirect effects. By selecting genetic variants specifically related to metabolites and conducting sensitivity analyses to exclude other potential pathways, we can substantiate the validity of this assumption.

### Data sources

The data for the 233 metabolites were obtained from the GWAS database (https://www.ebi.ac.uk/gwas/), covering 33 cohorts. After variant filtering and quality control, up to 13,389,637 imputed autosomal SNPs were included in the analysis, involving up to 136,016 participants. Data on coronary atherosclerosis were sourced from the R10 version of summary data from the FinnGen database (https://www.finngen.fi/fi), including 51,589 cases and 343,079 controls.

### Selection of instrumental variables

Using the “TwoSampleMR” package (version 0.5.7), we first selected instrumental variables (IVs) with *P* < 1 × 10^−5^, as referenced in relevant Mendelian randomization studies (7), to identify IVs significantly associated with the exposure factors. We set the clump parameter to TRUE to remove instrumental variables with linkage disequilibrium (LD), primarily measured using two parameters, *r*^2^ and kb, which were set to 0.001 and 10,000, respectively. This approach aimed to reduce collinearity and confounding bias, thereby enhancing the accuracy and reliability of causal inference analysis (8). Additionally, we calculated *F* values, defining *F*-statistics <10 as “weak IVs,” and excluded them.

### Statistical analysis

This study employed five methods—MR Egger, Weighted Median, Inverse Variance Weighted (IVW), Simple Mode, and Weighted Mode—to verify the causal relationship between 233 metabolites and susceptibility to coronary atherosclerosis. The results are primarily based on the IVW method, with other methods serving as supplementary approaches. Cochrane's *Q* test and its corresponding *p*-value were used to assess the heterogeneity of the IVs; when *P* < 0.05, the random-effects IVW was applied instead of the fixed-effects IVW (9). To account for potential horizontal pleiotropy, the MR Egger method was used, where a significant intercept term would indicate pleiotropy (10). Additionally, to identify and address outliers, we applied the MR-PRESSO test; any identified outliers were removed, and the MR causal estimates were recalculated (11, 12). Statistical significance was determined at *P* < 0.05.

## Results

### Causal relationship between 233 metabolites and coronary atherosclerosis

**Figure 2:**
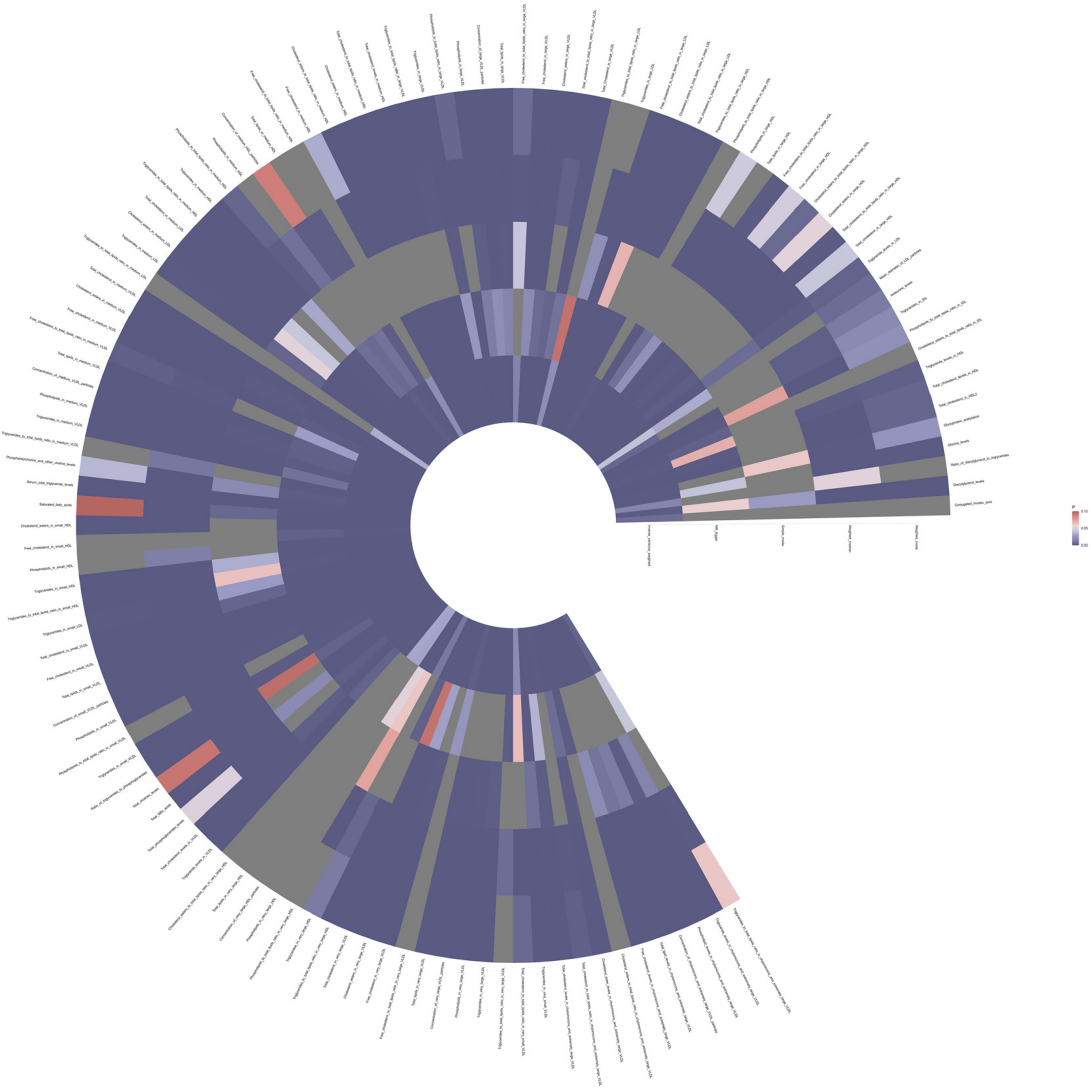
Full *P*-value results of five Mendelian randomized analyses of 118 metabolites associated with coronary atherosclerosis.

To investigate the impact of 233 metabolites on coronary atherosclerosis, we employed two-sample MR analysis, with IVW as the primary analysis method. The results revealed that 118 metabolites had a causal relationship with the susceptibility to coronary atherosclerosis (see Figure 2 and Supplementary Table 1).

### Metabolites increasing the risk of coronary atherosclerosis

Notably, among the 81 human metabolites identified as risk factors for the development of coronary atherosclerosis, the following were included:

Total fatty acids (TFA): The odds ratio (OR) for TFA was 1.40 (95% CI: 1.28–1.53, *P* < 0.001), indicating that a 1-unit increase in TFA levels is associated with a 40% higher risk of developing coronary atherosclerosis.

Saturated fatty acids (SFA): The OR for SFA was 1.44 (95% CI: 1.30–1.60, *P* < 0.001), indicating a 44% increase in the risk of coronary atherosclerosis per unit increase in SFA levels.

Serum total triglyceride levels: The OR for serum total triglycerides was 1.33 (95% CI: 1.22–1.46, *P* < 0.001), showing a 33% higher risk of coronary atherosclerosis with increased triglyceride levels.

Conjugated linoleic acid (CLA): The OR for CLA was 1.16 (95% CI: 1.04–1.30, *P* = 0.007), suggesting a 16% increase in the risk of coronary atherosclerosis per unit increase in CLA levels.

**Figure 3:**
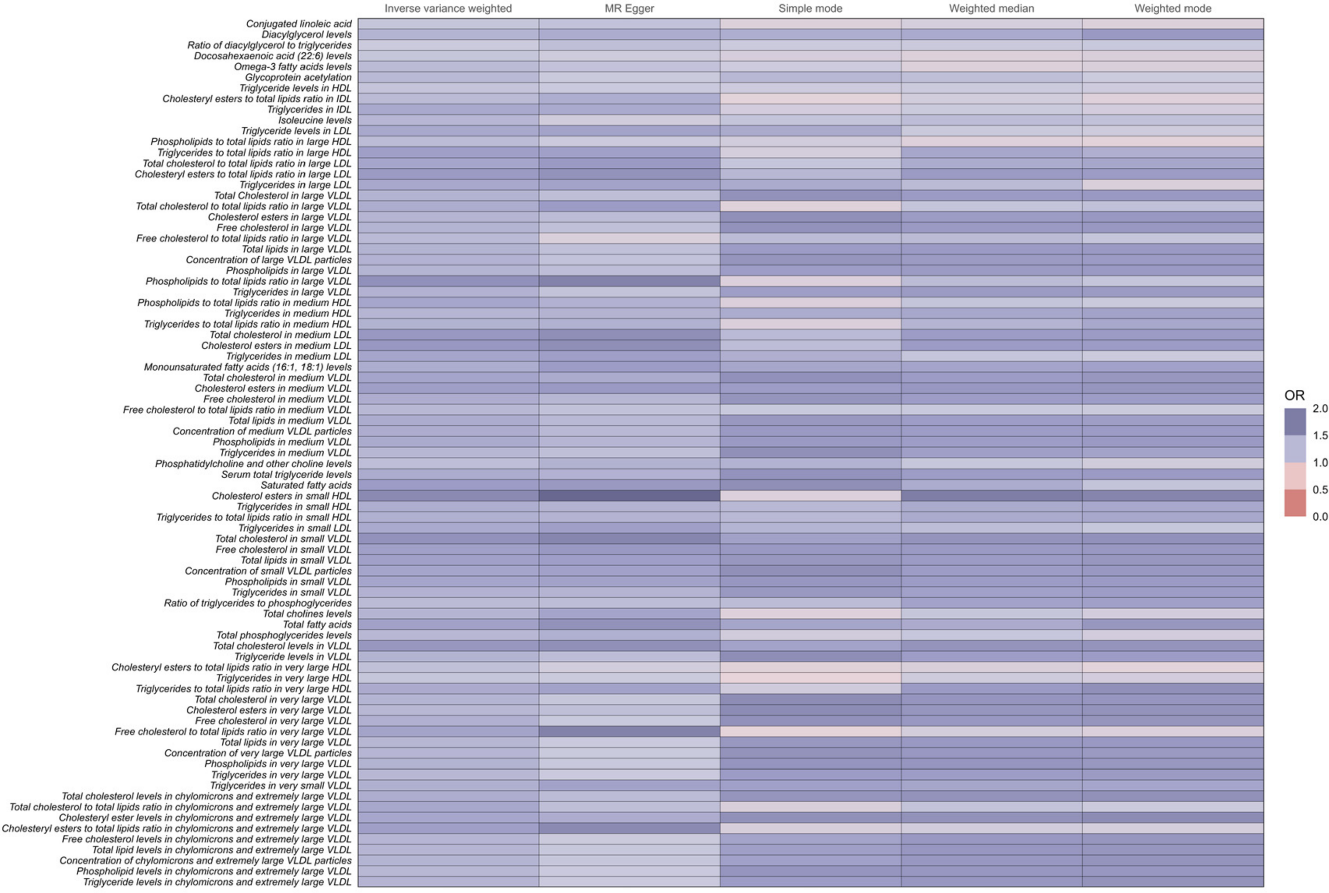
Full results of five Mendelian randomized OR values for 81metabolites associated with an increased risk of coronary atherosclerosis.

These metabolites are associated with an increased risk of developing coronary atherosclerosis (Figure 3 and Supplementary Table 1).

### Metabolites reducing the risk of coronary atherosclerosis

Conversely, among the 37 human metabolites were found to reduce the risk of coronary atherosclerosis, the following were included:

Cholesteryl esters to total lipids ratio in medium HDL: The OR for this ratio was 0.73 (95% CI: 0.67–0.78, *P* < 0.001), indicating that a 1-unit increase in this ratio reduces the risk of coronary atherosclerosis by 27%.

Cholesteryl esters to total lipids ratio in large HDL: The OR was 0.64 (95% CI: 0.58–0.71, *P* < 0.001), suggesting a 36% reduction in the risk of coronary atherosclerosis per unit increase in this ratio.

Total cholesterol to total lipids ratio in medium HDL: The OR was 0.71 (95% CI: 0.65–0.77, *P* < 0.001), demonstrating a 29% lower risk of coronary atherosclerosis with increased levels of this ratio.

Free cholesterol to total lipids ratio in large LDL: The OR was 0.70 (95% CI: 0.64–0.77, *P* < 0.001), reflecting a 30% reduced risk of coronary atherosclerosis per unit increase in this ratio.

Total cholesterol to total lipids ratio in large HDL: The OR was 0.70 (95% CI: 0.64–0.78, *P* < 0.001), suggesting a 30% reduction in the risk of coronary atherosclerosis.

**Figure 4:**
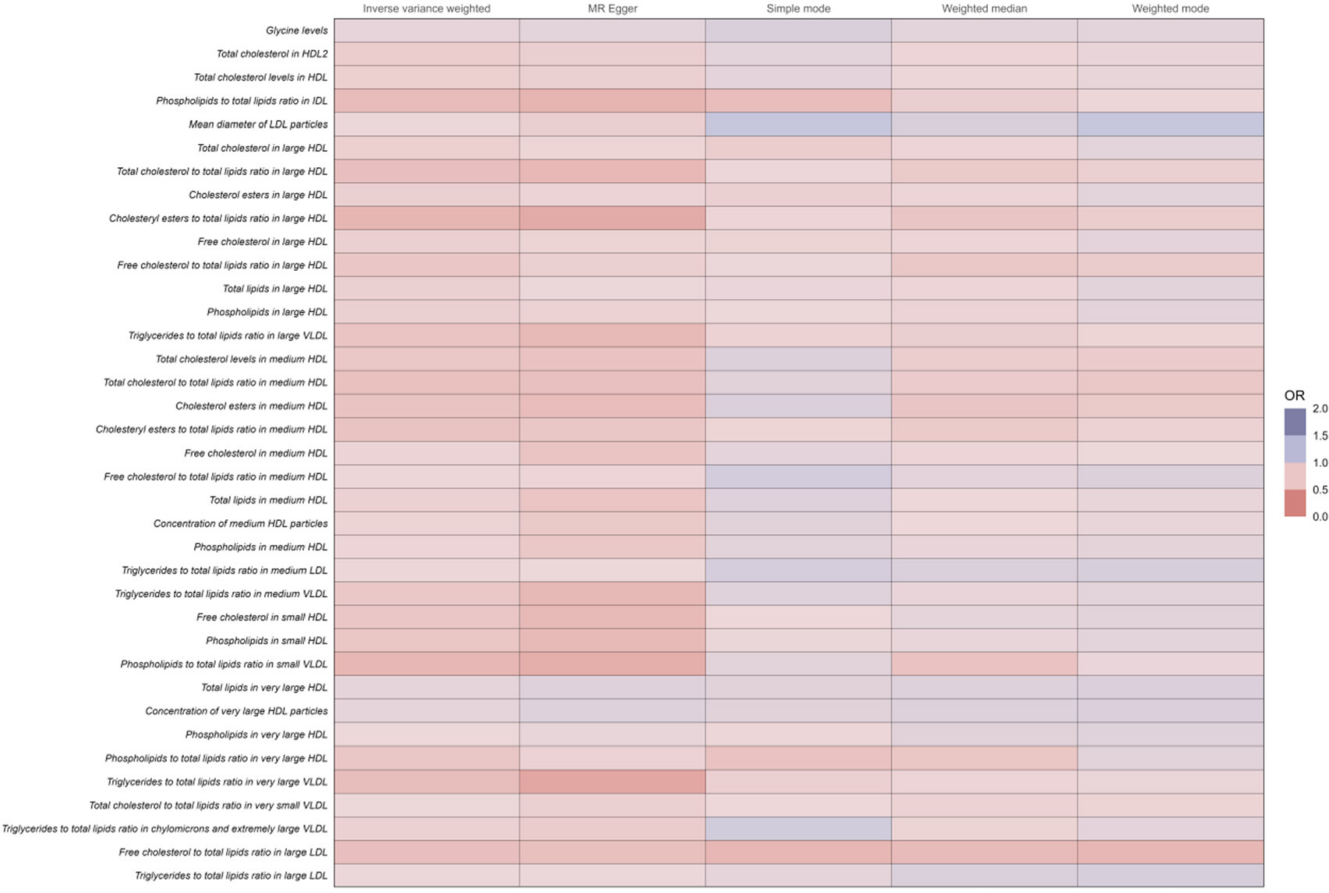
Full results of five Mendelian randomized analyses of OR values for 37 metabolites that reduce the risk of coronary atherosclerosis.

The increase in these ratios contributes to a decreased incidence of coronary atherosclerosis (Figure 4 and Supplementary Table 1).

### Sensitivity analysis

**Figure 5:**
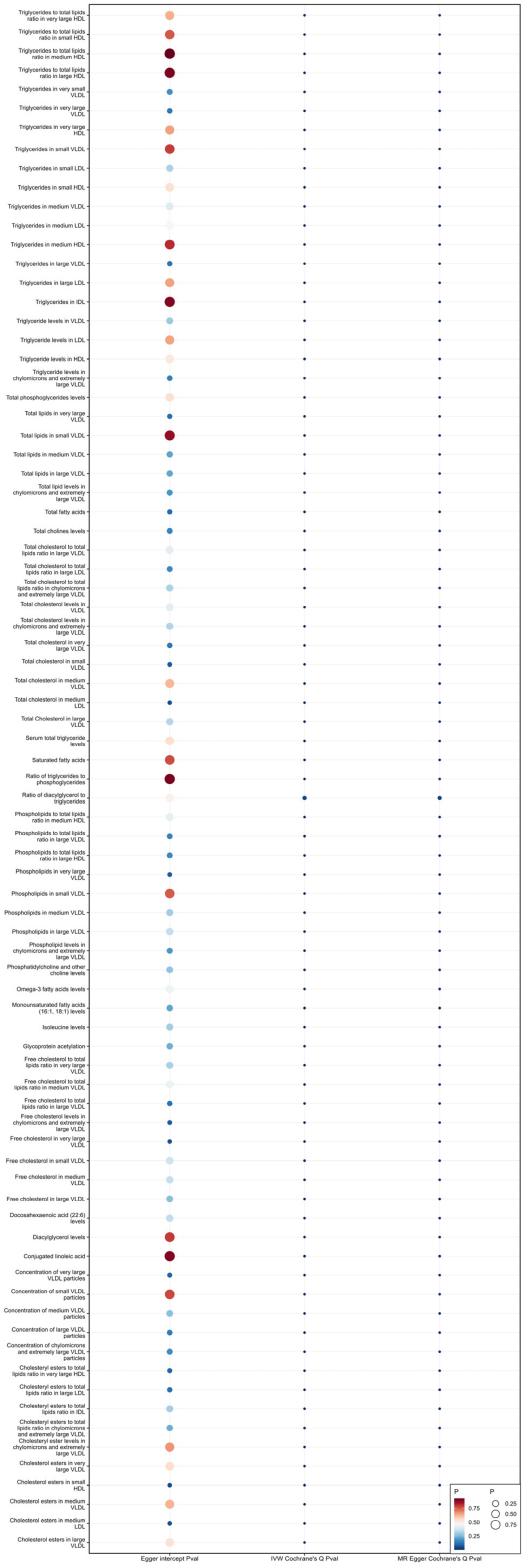
Heterogeneity and pleiotropy of 81 metabolites associated with increased risk of coronary atherosclerosis.

**Figure 6:**
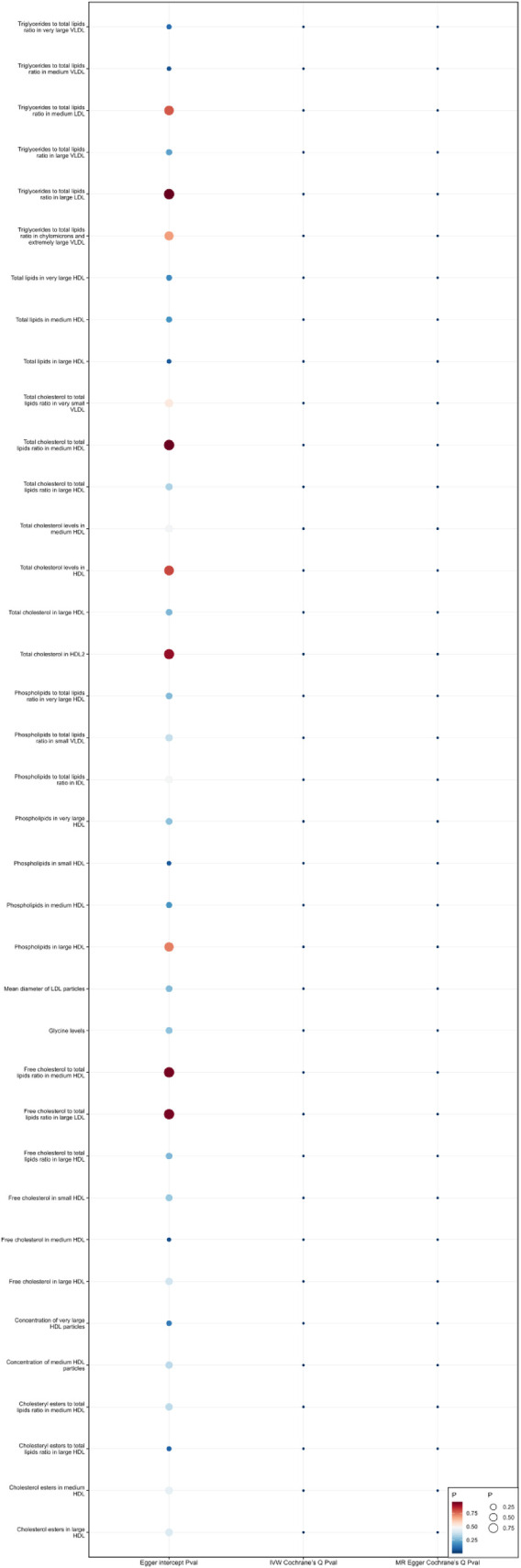
Heterogeneity and pleiotropy of 37 metabolites that reduce the risk of coronary atherosclerosis.

When using MR Egger, Weighted median, Simple mode, and Weighted mode for analysis, most results were consistent with those obtained using the IVW method (see Supplementary Table 1). In the MR-Egger analysis, there was no evidence of horizontal pleiotropy (see Supplementary Table 1, Figures 5, 6). Cochran's *Q* statistic indicated the presence of heterogeneity, so random effects IVW was used instead of fixed-effects IVW in these cases (see Supplementary Table 1, Figures 5, 6).

## Discussion

This study identified 118 metabolites with a causal relationship to the development of coronary atherosclerosis through Mendelian randomization. Among these, 81 metabolites were found to increase the risk of coronary atherosclerosis. The dysregulated metabolic pathways through which these metabolites contribute to atherosclerosis include lipid metabolism, oxidative stress, and inflammatory responses.

TFA are composed primarily of saturated fatty acids, trans fatty acids, polyunsaturated fatty acids, and other types of fatty acids. Among them, saturated and trans fatty acids are known to elevate low-density lipoprotein cholesterol (LDL-C) levels. A study conducted on a Nigerian population found that TFA is positively correlated with LDL-C (13). Elevated LDL-C is a major risk factor for the initiation and progression of atherosclerosis (14). LDL penetrates the vascular wall through the endothelium and becomes retained in the subendothelial layer, where it undergoes modification into oxidized LDL (ox-LDL). Macrophages engulf ox-LDL, forming foam cells, which proliferate and fuse, eventually forming the lipid core of atherosclerotic plaques (15). This aligns with the “response-to-retention” hypothesis (16), which highlights the retention and modification of LDL in the subendothelial space as critical to atherogenesis. Additionally, high levels of fatty acids, particularly unsaturated fatty acids, generate reactive oxygen species (ROS) during oxidation. On one hand, ROS impair vascular endothelial function by reducing nitric oxide (NO) bioavailability (17); on the other hand, they damage vascular endothelial cells and trigger inflammatory responses, accelerating vascular stiffening (18). For example, arachidonic acid and its downstream metabolite leukotriene B4(LTB4) are known mediators in atherosclerosis (19). Studies have shown that LTB4-induced neutrophil recruitment exacerbates plaque destabilization in endotoxemic contexts, suggesting that LTB4 could be a potential therapeutic target in the treatment of atherosclerosis (20). These findings highlighting the importance of TFA in coronary atherosclerosis.

The role of triglycerides (TG) in the pathogenesis of cardiovascular disease (CVD) has gained attention since Austin MA et al.'s meta-analysis in 1998 highlighted its association with CVD (21). Elevated TG levels lead to dyslipidemia, generating small dense LDL particles (sdLDL). These sdLDL particles, due to their smaller size, can more easily penetrate endothelial gaps and bind to proteoglycans within the arterial wall, where they accumulate. This prolonged retention makes sdLDL more prone to oxidative and other atherogenic modifications, further amplifying its pro-atherogenic properties (22). In addition, changes in oxLDL levels are correlated with changes in the number of sdLDL particles (23). Elevated oxLDL not only induces the expression of vascular cell adhesion molecule-1 (VCAM-1) in endothelial cells—exacerbating inflammation and promoting atherosclerosis (24)—but also triggers endothelial apoptosis, accelerating endothelial dysfunction and the progression of atherosclerosis (25). Clinical studies support these findings, showing that elevated TG levels are associated with lipid-rich plaques, thin-cap fibroatheromas (TCFAs), and a higher prevalence of macrophages in atherosclerotic plaques (26–29). Hypertriglyceridemia has also been suggested by some researchers as a significant contributor to recurrent atherosclerotic cardiovascular disease (ASCVD) events (30). Moreover, Arterial retention of triglyceride-rich lipoproteins (TRLs) and their remnants is linked to maladaptive responses central to plaque initiation and progression (31). Notably, research indicates that TRLs and their remnants are approximately four times more atherogenic than LDL (32). This mechanistic understanding of TG's role emphasizes its impact on the progression of coronary atherosclerosis.

SFA play a significant role in the pathogenesis of cardiovascular diseases (CVD) through multiple mechanisms, particularly by promoting inflammation and metabolic dysregulation. SFA activate inflammatory pathways via nuclear factor-kappa B (NF-κB), inducing inflammatory cytokines and contributing to vascular endothelial injury (33). Diets rich in SFAs can also cause metabolic imbalance and alterations in cellular signaling causing insulin resistance (34). Insulin resistance also disrupts systemic lipid metabolism, leading to dyslipidemia and the development of the well-known lipid triad: (1) elevated plasma triglyceride levels, (2) reduced high-density lipoprotein (HDL) levels, and (3) the presence of small dense low-density lipoprotein (sdLDL) particles. This lipid triad, along with endothelial dysfunction induced by aberrant insulin signaling, contributes to the formation of atherosclerotic plaques (35). Moreover, SFAs increase serum LDL levels, further promoting cardiovascular disease (36). Zong G et al. indicated that a high dietary intake of SFA correlates with increased coronary heart disease risk (37). Cross-sectional studies also link habitual SFA and trans fat intake with heightened subclinical atherosclerosis (38). These findings establish a close relationship between SFA intake and coronary atherosclerosis.

CLA primarily found in the milk and meat of ruminant animals (39), includes over 28 isomers. In natural products, the predominant isomer (≥80% of total CLA) is cis-9, trans-11, while commercial preparations vary in isomer composition (40). Animal studies have produced conflicting findings, with some suggesting CLA's protective effects against atherosclerosis (41–44), while others indicate no benefit, or even a pro-atherosclerotic effect of trans10, cis12-CLA (45). In human studies, a randomized double-blind study involving healthy young men showed that a CLA diet increased levels of the lipid peroxidation marker 8-iso-prostaglandin F2α (8-iso-PGF2α), showing an 83% elevation compared to the control group (46), indicating heightened oxidative stress (47). The increased oxidative stress induced by CLA, as evidenced by elevated 8-iso-PGF2α, reduces NO bioavailability, contributing to endothelial dysfunction and vascular aging (17). Another study with postmenopausal women demonstrated that oil containing trans10, cis12-CLA increased lipid peroxidation, thereby promoting atherosclerosis development (48). Increased lipid peroxidation disrupts normal lipid metabolism, leading to the accumulation of oxLDL within the arterial intima and further advancing atherosclerosis (25). Future research should focus on isomer-specific effects of CLA and its role in cardiovascular health to inform dietary recommendations and therapeutic interventions.

In summary, our findings emphasize the role of lipid metabolism, oxidative stress, and inflammation as key dysregulated metabolic pathways through which these metabolites contribute to the pathogenesis of coronary atherosclerosis. The intricate interplay of these pathways suggests that targeting these metabolic disturbances may be effective in mitigating coronary atherosclerosis risk.

This study has several limitations. First, Although we have made every effort to minimize confounding factors and reduce the influence of pleiotropy at the genetic level, unmeasured confounding factors may still exist. For example, environmental factors and lifestyle variables, which are not fully considered in genetic analyses, may lead to potential bias. Additionally, pleiotropy remains a challenge, as genetic variants may influence the outcome through multiple biological pathways unrelated to the metabolites studied, potentially impacting the reliability of the causal estimates. Second, this study focuses primarily on the FinnGen database, which may limit the generalizability of the findings to other populations. Future studies incorporating diverse populations and addressing these confounders more rigorously will be necessary to validate our findings.

In conclusion, this study conducted a two-sample Mendelian randomization analysis to explore the causal relationships between 233 metabolites and coronary atherosclerosis based on the summary statistics of a GWAS of 233 metabolites and the FinnGen R10 release data. These findings may help elucidate the impact of metabolites on coronary atherosclerosis and inspire the development of precision medicine in this field.

## Data Availability

Publicly available datasets were analyzed in this study. This data can be found here: 233 metabolites (GCST90301941-GCST90302173) available at https://www.ebi.ac.uk/gwas/. Coronary atherosclerosis: summary statistics can be accessed at https://storage.googleapis.com/finngen-public-data-r10/summary_stats/finngen_R10_I9_CORATHER.gz
