# Supplementary material for: The causal relationship between 233 metabolites and coronary atherosclerosis: a Mendelian randomization study
Source: Front Cardiovasc Med. 2024 Dec 12;11:1439699. doi: 10.3389/fcvm.2024.1439699 (PMC11669696; doi:10.3389/fcvm.2024.1439699)
Supplement: Supplementary file 1 [file Table1.pdf]

Supplementary Table 1 Full results of 118 metabolites associated with coronary atherosclerosis

Metabolites	Method	P-value	OR(95%CI)	Heterogeneity		Pleiotropy
				MR Egger Cochrane's Q P-value	IVW Cochrane's Q P-value	Egger intercept P-value
Conjugated linoleic acid	MR Egger	0.2799	1.1876(0.8734-1.6148)			
Conjugated linoleic acid	Weighted median	0.4716	1.0506(0.9185-1.2017)			
Conjugated linoleic acid	Inverse variance weighted	0.0069	1.1646(1.0428-1.3006)	<0.001	<0.001	0.8942
Conjugated linoleic acid	Simple mode	0.9669	0.9941(0.7547-1.3096)			
Conjugated linoleic acid	Weighted mode	0.9673	0.9941(0.7520-1.3142)			
Diacylglycerol levels	MR Egger	0.0598	1.3300(0.9934-1.7808)			
Diacylglycerol levels	Weighted median	<0.001	1.3440(1.2160-1.4855)			
Diacylglycerol levels	Inverse variance weighted	<0.001	1.2862(1.1101-1.4903)	<0.001	<0.001	0.7947
Diacylglycerol levels	Simple mode	0.0268	1.3169(1.0376-1.6713)			
Diacylglycerol levels	Weighted mode	<0.001	1.4722(1.2706-1.7057)			
Ratio of diacylglycerol to triglycerides	MR Egger	0.1073	1.2098(0.9648-1.5171)			
Ratio of diacylglycerol to triglycerides	Weighted median	0.0521	1.1253(0.9989-1.2677)			
Ratio of diacylglycerol to triglycerides	Inverse variance weighted	0.0158	1.1232(1.0220-1.2344)	0.0389	0.0427	0.4827
Ratio of diacylglycerol to triglycerides	Simple mode	0.3411	1.1187(0.8905-1.4053)			
Ratio of diacylglycerol to triglycerides	Weighted mode	0.201	1.1407(0.9354-1.3910)			
Docosahexaenoic acid (22:6) levels	MR Egger	0.1737	1.1050(0.9579-1.2747)			
Docosahexaenoic acid (22:6) levels	Weighted median	0.8593	0.9938(0.9283-1.0640)			
Docosahexaenoic acid (22:6) levels	Inverse variance weighted	0.0012	1.1642(1.0618-1.2766)	<0.001	<0.001	0.3507
Docosahexaenoic acid (22:6) levels	Simple mode	0.7366	1.0416(0.8218-1.3202)			
Docosahexaenoic acid (22:6) levels	Weighted mode	0.5442	1.0218(0.9533-1.0952)			
Omega-3 fatty acids levels	MR Egger	0.0126	1.1843(1.0392-1.3496)			
Omega-3 fatty acids levels	Weighted median	0.9112	0.9964(0.9359-1.0609)			
Omega-3 fatty acids levels	Inverse variance weighted	<0.001	1.2301(1.1262-1.3437)	<0.001	<0.001	0.4408

Metabolites	Method	P-value	OR(95%CI)	Heterogeneity		Pleiotropy
				MR Egger Cochrane’s Q P-value	IVW Cochrane’s Q P-value	Egger intercept P-value
Omega-3 fatty acids levels	Simple mode	0.431	1.1083(0.8588-1.4302)			
Omega-3 fatty acids levels	Weighted mode	0.624	1.0163(0.9530-1.0838)			
Glycine levels	MR Egger	0.0409	0.9355(0.8785-0.9963)			
Glycine levels	Weighted median	<0.001	0.9376(0.9053-0.9710)			
Glycine levels	Inverse variance weighted	<0.001	0.9155(0.8716-0.9615)	<0.001	<0.001	0.2836
Glycine levels	Simple mode	0.7612	1.0258(0.8707-1.2085)			
Glycine levels	Weighted mode	<0.001	0.9354(0.9026-0.9695)			
Glycoprotein acetylation	MR Egger	0.2429	1.1284(0.9222-1.3807)			
Glycoprotein acetylation	Weighted median	<0.001	1.2084(1.0934-1.3354)			
Glycoprotein acetylation	Inverse variance weighted	<0.001	1.2438(1.1008-1.4054)	<0.001	<0.001	0.2378
Glycoprotein acetylation	Simple mode	0.0621	1.2566(0.9908-1.5937)			
Glycoprotein acetylation	Weighted mode	0.0241	1.1189(1.0161-1.2321)			
Total cholesterol in HDL2	MR Egger	<0.001	0.7992(0.7128-0.8961)			
Total cholesterol in HDL2	Weighted median	<0.001	0.8457(0.7800-0.9170)			
Total cholesterol in HDL2	Inverse variance weighted	<0.001	0.7947(0.7431-0.8499)	<0.001	<0.001	0.9035
Total cholesterol in HDL2	Simple mode	0.5846	0.9412(0.7578-1.1690)			
Total cholesterol in HDL2	Weighted mode	0.0044	0.8908(0.8235-0.9636)			
Total cholesterol levels in HDL	MR Egger	<0.001	0.8125(0.7283-0.9064)			
Total cholesterol levels in HDL	Weighted median	<0.001	0.8477(0.7821-0.9189)			
Total cholesterol levels in HDL	Inverse variance weighted	<0.001	0.8037(0.7539-0.8568)	<0.001	<0.001	0.8106
Total cholesterol levels in HDL	Simple mode	0.5125	0.9328(0.7580-1.1481)			
Total cholesterol levels in HDL	Weighted mode	0.0043	0.9038(0.8439-0.9680)			
Triglyceride levels in HDL	MR Egger	0.0698	1.1339(0.9911-1.2973)			
Triglyceride levels in HDL	Weighted median	0.0012	1.1225(1.0469-1.2036)			
Triglyceride levels in HDL	Inverse variance weighted	<0.001	1.1720(1.0685-1.2855)	<0.001	<0.001	0.5084
Triglyceride levels in HDL	Simple mode	0.3617	1.1092(0.8885-1.3847)			

Metabolites	Method	P-value	OR(95%CI)	Heterogeneity		Pleiotropy
				MR Egger Cochrane's Q P-value	IVW Cochrane's Q P-value	Egger intercept P-value
Triglyceride levels in HDL	Weighted mode	0.0024	1.1092(1.0389-1.1842)			
Cholesteryl esters to total lipids ratio in IDL	MR Egger	0.0025	1.3177(1.1068-1.5688)			
Cholesteryl esters to total lipids ratio in IDL	Weighted median	0.2334	1.0702(0.9572-1.1964)			
Cholesteryl esters to total lipids ratio in IDL	Inverse variance weighted	<0.001	1.2319(1.0947-1.3863)	<0.001	<0.001	0.3065
Cholesteryl esters to total lipids ratio in IDL	Simple mode	0.6282	0.9404(0.7338-1.2051)			
Cholesteryl esters to total lipids ratio in IDL	Weighted mode	0.8162	0.9705(0.7544-1.2484)			
Phospholipids to total lipids ratio in IDL	MR Egger	<0.001	0.6315(0.4936-0.8079)			
Phospholipids to total lipids ratio in IDL	Weighted median	<0.001	0.8010(0.7254-0.8845)			
Phospholipids to total lipids ratio in IDL	Inverse variance weighted	<0.001	0.6811(0.5949-0.7797)	<0.001	<0.001	0.4735
Phospholipids to total lipids ratio in IDL	Simple mode	0.0756	0.6869(0.4559-1.0348)			
Phospholipids to total lipids ratio in IDL	Weighted mode	0.0219	0.8512(0.7433-0.9748)			
Triglycerides in IDL	MR Egger	<0.001	1.3433(1.2088-1.4928)			
Triglycerides in IDL	Weighted median	0.0017	1.1272(1.0458-1.2148)			
Triglycerides in IDL	Inverse variance weighted	<0.001	1.3507(1.2584-1.4497)	<0.001	<0.001	0.8913
Triglycerides in IDL	Simple mode	0.5524	1.0763(0.8450-1.3708)			
Triglycerides in IDL	Weighted mode	0.0187	1.0763(1.0129-1.1436)			
Isoleucine levels	MR Egger	0.6486	1.0815(0.7731-1.5128)			
Isoleucine levels	Weighted median	0.0019	1.2189(1.0755-1.3815)			
Isoleucine levels	Inverse variance weighted	0.0047	1.2628(1.0743-1.4844)	<0.001	<0.001	0.3045
Isoleucine levels	Simple mode	0.2153	1.1768(0.9113-1.5196)			
Isoleucine levels	Weighted mode	0.0117	1.1939(1.0431-1.3664)			
Mean diameter of LDL particles	MR Egger	0.0336	0.8000(0.6532-0.9798)			
Mean diameter of LDL particles	Weighted median	0.6299	1.0291(0.9158-1.1563)			
Mean diameter of LDL particles	Inverse variance weighted	0.0403	0.8749(0.7701-0.9941)	<0.001	<0.001	0.2683

Metabolites	Method	P-value	OR(95%CI)	Heterogeneity		Pleiotropy
				MR Egger Cochrane' s Q P-value	IVW Cochrane' s Q P-value	Egger intercept P-value
Mean diameter of LDL particles	Simple mode	0.3491	1.1600(0.8516-1.5800)			
Mean diameter of LDL particles	Weighted mode	0.0066	1.1600(1.0449-1.2878)			
Triglyceride levels in LDL	MR Egger	<0.001	1.3802(1.2296-1.5491)			
Triglyceride levels in LDL	Weighted median	0.0011	1.1308(1.0505-1.2173)			
Triglyceride levels in LDL	Inverse variance weighted	<0.001	1.3526(1.2525-1.4607)	<0.001	<0.001	0.6463
Triglyceride levels in LDL	Simple mode	0.0065	1.3349(1.0875-1.6386)			
Triglyceride levels in LDL	Weighted mode	0.0033	1.1043(1.0346-1.1786)			
Total cholesterol in large HDL	MR Egger	0.0015	0.8422(0.7588-0.9348)			
Total cholesterol in large HDL	Weighted median	<0.001	0.8444(0.7791-0.9150)			
Total cholesterol in large HDL	Inverse variance weighted	<0.001	0.8033(0.7533-0.8566)	<0.001	<0.001	0.2609
Total cholesterol in large HDL	Simple mode	0.1043	0.7958(0.6050-1.0468)			
Total cholesterol in large HDL	Weighted mode	0.0423	0.9364(0.8793-0.9972)			
Total cholesterol to total lipids ratio in large HDL	MR Egger	<0.001	0.6584(0.5591-0.7754)			
Total cholesterol to total lipids ratio in large HDL	Weighted median	<0.001	0.7753(0.7060-0.8514)			
Total cholesterol to total lipids ratio in large HDL	Inverse variance weighted	<0.001	0.7031(0.6372-0.7757)	<0.001	<0.001	0.3266
Total cholesterol to total lipids ratio in large HDL	Simple mode	0.3693	0.8691(0.6405-1.1793)			
Total cholesterol to total lipids ratio in large HDL	Weighted mode	<0.001	0.8061(0.7148-0.9091)			
Cholesterol esters in	MR Egger	<0.001	0.8339(0.7519-0.9250)			

Metabolites	Method	P-value	OR(95%CI)	Heterogeneity		Pleiotropy
				MR Egger Cochrane's Q P-value	IVW Cochrane's Q P-value	Egger intercept P-value
large HDL						
Cholesterol esters in large HDL	Weighted median	<0.001	0.8451(0.7803-0.9154)			
Cholesterol esters in large HDL	Inverse variance weighted	<0.001	0.8059(0.7562-0.8589)	<0.001	<0.001	0.4132
Cholesterol esters in large HDL	Simple mode	0.1208	0.8113(0.6237-1.0552)			
Cholesterol esters in large HDL	Weighted mode	0.0527	0.9355(0.8749-1.0003)			
Cholesteryl esters to total lipids ratio in large HDL	MR Egger	<0.001	0.5678(0.4771-0.6756)			
Cholesteryl esters to total lipids ratio in large HDL	Weighted median	<0.001	0.7419(0.6693-0.8224)			
Cholesteryl esters to total lipids ratio in large HDL	Inverse variance weighted	<0.001	0.6407(0.5761-0.7125)	<0.001	<0.001	0.0894
Cholesteryl esters to total lipids ratio in large HDL	Simple mode	0.4115	0.8434(0.5624-1.2646)			
Cholesteryl esters to total lipids ratio in large HDL	Weighted mode	0.0059	0.7925(0.6736-0.9323)			
Free cholesterol in large HDL	MR Egger	<0.001	0.8374(0.7596-0.9231)			
Free cholesterol in large HDL	Weighted median	<0.001	0.8432(0.7779-0.9139)			
Free cholesterol in large HDL	Inverse variance weighted	<0.001	0.8096(0.7624-0.8597)	<0.001	<0.001	0.3894
Free cholesterol in large HDL	Simple mode	0.1813	0.8363(0.6442-1.0857)			
Free cholesterol in large HDL	Weighted mode	0.0464	0.9304(0.8672-0.9983)			
Free cholesterol to total lipids ratio in large HDL	MR Egger	0.0223	0.8141(0.6839-0.9690)			
Free cholesterol to total lipids ratio in large HDL	Weighted median	<0.001	0.7579(0.6887-0.8340)			
Free cholesterol to total lipids ratio in large HDL	Inverse variance weighted	<0.001	0.7508(0.6774-0.8322)	<0.001	<0.001	0.2619

Metabolites	Method	P-value	OR(95%CI)	Heterogeneity		Pleiotropy
				MR Egger Cochrane’s Q P-value	IVW Cochrane’s Q P-value	Egger intercept P-value
Free cholesterol to total lipids ratio in large HDL	Simple mode	0.4357	0.8760(0.6286-1.2207)			
Free cholesterol to total lipids ratio in large HDL	Weighted mode	<0.001	0.7870(0.6861-0.9027)			
Total lipids in large HDL	MR Egger	0.0083	0.8746(0.7927-0.9650)			
Total lipids in large HDL	Weighted median	<0.001	0.8408(0.7826-0.9034)			
Total lipids in large HDL	Inverse variance weighted	<0.001	0.8149(0.7661-0.8667)	<0.001	<0.001	0.0732
Total lipids in large HDL	Simple mode	0.32	0.8936(0.7163-1.1147)			
Total lipids in large HDL	Weighted mode	0.1277	0.9474(0.8841-1.0153)			
Phospholipids in large HDL	MR Egger	<0.001	0.8253(0.7482-0.9104)			
Phospholipids in large HDL	Weighted median	<0.001	0.8343(0.7751-0.8980)			
Phospholipids in large HDL	Inverse variance weighted	<0.001	0.8139(0.7669-0.8638)	<0.001	<0.001	0.7269
Phospholipids in large HDL	Simple mode	0.2178	0.8666(0.6907-1.0872)			
Phospholipids in large HDL	Weighted mode	0.0444	0.9308(0.8684-0.9976)			
Phospholipids to total lipids ratio in large HDL	MR Egger	0.2027	1.1191(0.9421-1.3293)			
Phospholipids to total lipids ratio in large HDL	Weighted median	0.4622	0.9693(0.8919-1.0534)			
Phospholipids to total lipids ratio in large HDL	Inverse variance weighted	<0.001	1.2261(1.0920-1.3767)	<0.001	<0.001	0.1639
Phospholipids to total lipids ratio in large HDL	Simple mode	0.5978	1.0804(0.8113-1.4389)			
Phospholipids to total lipids ratio in large HDL	Weighted mode	0.1082	0.9431(0.8784-1.0124)			
Triglycerides to total lipids ratio in large HDL	MR Egger	<0.001	1.4076(1.2375-1.6011)			
Triglycerides to total lipids ratio in large HDL	Weighted median	<0.001	1.3646(1.2464-1.4940)			
Triglycerides to total lipids ratio in large HDL	Inverse variance weighted	<0.001	1.4169(1.3082-1.5346)	<0.001	<0.001	0.8978
Triglycerides to total lipids ratio in large HDL	Simple mode	0.6163	1.0603(0.8438-1.3323)			
Triglycerides to total lipids ratio in large HDL	Weighted mode	<0.001	1.3069(1.1914-1.4335)			

Metabolites	Method	P-value	OR(95%CI)	Heterogeneity		Pleiotropy
				MR Egger Cochrane' s Q P-value	IVW Cochrane' s Q P-value	Egger intercept P-value
Total cholesterol to total lipids ratio in large LDL	MR Egger	<0.001	1.4602(1.2928-1.6492)			
Total cholesterol to total lipids ratio in large LDL	Weighted median	<0.001	1.3290(1.2348-1.4304)			
Total cholesterol to total lipids ratio in large LDL	Inverse variance weighted	<0.001	1.3734(1.2600-1.4970)	<0.001	<0.001	0.1667
Total cholesterol to total lipids ratio in large LDL	Simple mode	0.1308	1.1703(0.9556-1.4334)			
Total cholesterol to total lipids ratio in large LDL	Weighted mode	<0.001	1.3633(1.2477-1.4895)			
Cholesteryl esters to total lipids ratio in large LDL	MR Egger	<0.001	1.5366(1.3936-1.6942)			
Cholesteryl esters to total lipids ratio in large LDL	Weighted median	<0.001	1.4456(1.3394-1.5601)			
Cholesteryl esters to total lipids ratio in large LDL	Inverse variance weighted	<0.001	1.4535(1.3560-1.5580)	<0.001	<0.001	0.1173
Cholesteryl esters to total lipids ratio in large LDL	Simple mode	0.0683	1.2527(0.9852-1.5927)			
Cholesteryl esters to total lipids ratio in large LDL	Weighted mode	<0.001	1.4383(1.3382-1.5458)			
Free cholesterol to total lipids ratio in large LDL	MR Egger	<0.001	0.7058(0.6055-0.8227)			
Free cholesterol to total lipids ratio in large LDL	Weighted median	<0.001	0.6748(0.6216-0.7326)			
Free cholesterol to total lipids ratio in large LDL	Inverse variance weighted	<0.001	0.7025(0.6384-0.7731)	<0.001	<0.001	0.9395
Free cholesterol to total lipids ratio in large LDL	Simple mode	<0.001	0.6495(0.5284-0.7984)			
Free cholesterol to total lipids ratio in large LDL	Weighted mode	<0.001	0.6495(0.6001-0.7030)			

Metabolites	Method	P-value	OR(95%CI)	Heterogeneity		Pleiotropy
				MR Egger Cochrane’s Q P-value	IVW Cochrane’s Q P-value	Egger intercept P-value
Triglycerides in large LDL	MR Egger	<0.001	1.3901(1.2282-1.5735)			
Triglycerides in large LDL	Weighted median	<0.001	1.2311(1.1309-1.3401)			
Triglycerides in large LDL	Inverse variance weighted	<0.001	1.3602(1.2558-1.4734)	<0.001	<0.001	0.6527
Triglycerides in large LDL	Simple mode	0.0213	1.3390(1.0471-1.7122)			
Triglycerides in large LDL	Weighted mode	0.4397	1.0292(0.9569-1.1069)			
Triglycerides to total lipids ratio in large LDL	MR Egger	0.0934	0.8699(0.7404-1.0220)			
Triglycerides to total lipids ratio in large LDL	Weighted median	0.8244	1.0089(0.9327-1.0913)			
Triglycerides to total lipids ratio in large LDL	Inverse variance weighted	0.0209	0.8723(0.7767-0.9796)	<0.001	<0.001	0.9615
Triglycerides to total lipids ratio in large LDL	Simple mode	0.4977	0.9184(0.7188-1.1735)			
Triglycerides to total lipids ratio in large LDL	Weighted mode	0.3999	1.0368(0.9535-1.1273)			
Total Cholesterol in large VLDL	MR Egger	0.0091	1.2158(1.0520-1.4052)			
Total Cholesterol in large VLDL	Weighted median	<0.001	1.4475(1.3431-1.5601)			
Total Cholesterol in large VLDL	Inverse variance weighted	<0.001	1.2840(1.1678-1.4118)	<0.001	<0.001	0.3298
Total Cholesterol in large VLDL	Simple mode	<0.001	1.5438(1.2519-1.9037)			
Total Cholesterol in large VLDL	Weighted mode	<0.001	1.4695(1.3717-1.5744)			
Total cholesterol to total lipids ratio in large VLDL	MR Egger	0.0039	1.4365(1.1320-1.8230)			
Total cholesterol to total lipids ratio in large VLDL	Weighted median	0.003	1.1745(1.0561-1.3061)			
Total cholesterol to total lipids ratio in large VLDL	Inverse variance weighted	<0.001	1.3246(1.1539-1.5206)	<0.001	<0.001	0.4154
Total cholesterol to	Simple mode	0.7329	0.9589(0.7543-1.2190)			

Metabolites	Method	P-value	OR(95%CI)	Heterogeneity		Pleiotropy
				MR Egger Cochrane’s Q P-value	IVW Cochrane’s Q P-value	Egger intercept P-value
total lipids ratio in large VLDL						
Total cholesterol to total lipids ratio in large VLDL	Weighted mode	<0.001	1.1926(1.0987-1.2945)			
Cholesterol esters in large VLDL	MR Egger	0.0062	1.2281(1.0624-1.4198)			
Cholesterol esters in large VLDL	Weighted median	<0.001	1.4440(1.3404-1.5557)			
Cholesterol esters in large VLDL	Inverse variance weighted	<0.001	1.2718(1.1569-1.3981)	<0.001	<0.001	0.5333
Cholesterol esters in large VLDL	Simple mode	<0.001	1.5669(1.2594-1.9494)			
Cholesterol esters in large VLDL	Weighted mode	<0.001	1.4789(1.3847-1.5797)			
Free cholesterol in large VLDL	MR Egger	0.0193	1.2034(1.0326-1.4023)			
Free cholesterol in large VLDL	Weighted median	<0.001	1.4605(1.3469-1.5836)			
Free cholesterol in large VLDL	Inverse variance weighted	<0.001	1.2854(1.1635-1.4201)	<0.001	<0.001	0.2679
Free cholesterol in large VLDL	Simple mode	<0.001	1.5393(1.2539-1.8896)			
Free cholesterol in large VLDL	Weighted mode	<0.001	1.4783(1.3736-1.5909)			
Free cholesterol to total lipids ratio in large VLDL	MR Egger	0.9636	1.0073(0.7374-1.3761)			
Free cholesterol to total lipids ratio in large VLDL	Weighted median	<0.001	1.2337(1.0989-1.3852)			
Free cholesterol to total lipids ratio in large VLDL	Inverse variance weighted	0.0148	1.2402(1.0430-1.4747)	<0.001	<0.001	0.122
Free cholesterol to total lipids ratio in large VLDL	Simple mode	0.0415	1.2284(1.0114-1.4920)			
Free cholesterol to total lipids ratio in large VLDL	Weighted mode	0.0068	1.1696(1.0474-1.3061)			

Metabolites	Method	P-value	OR(95%CI)	Heterogeneity		Pleiotropy
				MR Egger Cochrane's Q P-value	IVW Cochrane's Q P-value	Egger intercept P-value
Total lipids in large VLDL	MR Egger	0.0159	1.1984(1.0364-1.3858)			
Total lipids in large VLDL	Weighted median	<0.001	1.4514(1.3382-1.5741)			
Total lipids in large VLDL	Inverse variance weighted	<0.001	1.2836(1.1665-1.4125)	<0.001	<0.001	0.2209
Total lipids in large VLDL	Simple mode	<0.001	1.4400(1.1882-1.7451)			
Total lipids in large VLDL	Weighted mode	<0.001	1.4546(1.3598-1.5561)			
Concentration of large VLDL particles	MR Egger	0.0206	1.1869(1.0285-1.3697)			
Concentration of large VLDL particles	Weighted median	<0.001	1.4534(1.3499-1.5649)			
Concentration of large VLDL particles	Inverse variance weighted	<0.001	1.2881(1.1720-1.4158)	<0.001	<0.001	0.1396
Concentration of large VLDL particles	Simple mode	<0.001	1.5125(1.2454-1.8368)			
Concentration of large VLDL particles	Weighted mode	<0.001	1.4525(1.3590-1.5524)			
Phospholipids in large VLDL	MR Egger	0.0124	1.2104(1.0444-1.4028)			
Phospholipids in large VLDL	Weighted median	<0.001	1.4534(1.3466-1.5686)			
Phospholipids in large VLDL	Inverse variance weighted	<0.001	1.2765(1.1591-1.4057)	<0.001	<0.001	0.3525
Phospholipids in large VLDL	Simple mode	0.0014	1.4252(1.1526-1.7623)			
Phospholipids in large VLDL	Weighted mode	<0.001	1.4563(1.3537-1.5666)			
Phospholipids to total lipids ratio in large VLDL	MR Egger	<0.001	1.7016(1.4454-2.0033)			
Phospholipids to total lipids ratio in large VLDL	Weighted median	<0.001	1.2285(1.1087-1.3614)			
Phospholipids to total lipids ratio in large VLDL	Inverse variance weighted	<0.001	1.5499(1.3976-1.7187)	<0.001	<0.001	0.1524
Phospholipids to total	Simple mode	0.9982	1.0002(0.8172-1.2242)			

Metabolites	Method	P-value	OR(95%CI)	Heterogeneity		Pleiotropy
				MR Egger Cochrane's Q P-value	IVW Cochrane's Q P-value	Egger intercept P-value
lipids ratio in large VLDL						
Phospholipids to total lipids ratio in large VLDL	Weighted mode	0.0046	1.1703(1.0525-1.3013)			
Triglycerides in large VLDL	MR Egger	0.0254	1.1812(1.0225-1.3647)			
Triglycerides in large VLDL	Weighted median	<0.001	1.4544(1.3500-1.5669)			
Triglycerides in large VLDL	Inverse variance weighted	<0.001	1.2880(1.1712-1.4163)	<0.001	<0.001	0.1226
Triglycerides in large VLDL	Simple mode	<0.001	1.4242(1.1610-1.7471)			
Triglycerides in large VLDL	Weighted mode	<0.001	1.4542(1.3554-1.5600)			
Triglycerides to total lipids ratio in large VLDL	MR Egger	<0.001	0.6617(0.5424-0.8073)			
Triglycerides to total lipids ratio in large VLDL	Weighted median	<0.001	0.8005(0.7112-0.9010)			
Triglycerides to total lipids ratio in large VLDL	Inverse variance weighted	<0.001	0.7315(0.6482-0.8256)	<0.001	<0.001	0.2174
Triglycerides to total lipids ratio in large VLDL	Simple mode	0.1338	0.8243(0.6420-1.0584)			
Triglycerides to total lipids ratio in large VLDL	Weighted mode	<0.001	0.8414(0.7732-0.9156)			
Total cholesterol levels in medium HDL	MR Egger	<0.001	0.7233(0.6224-0.8405)			
Total cholesterol levels in medium HDL	Weighted median	<0.001	0.7939(0.7298-0.8637)			
Total cholesterol levels in medium HDL	Inverse variance weighted	<0.001	0.7572(0.6948-0.8252)	<0.001	<0.001	0.4673
Total cholesterol levels in medium HDL	Simple mode	0.9337	0.9907(0.7946-1.2351)			
Total cholesterol levels in medium HDL	Weighted mode	<0.001	0.7768(0.6895-0.8752)			

Metabolites	Method	P-value	OR(95%CI)	Heterogeneity		Pleiotropy
				MR Egger Cochrane’s Q P-value	IVW Cochrane’s Q P-value	Egger intercept P-value
Total cholesterol to total lipids ratio in medium HDL	MR Egger	<0.001	0.7097(0.6231-0.8083)			
Total cholesterol to total lipids ratio in medium HDL	Weighted median	<0.001	0.7782(0.7183-0.8430)			
Total cholesterol to total lipids ratio in medium HDL	Inverse variance weighted	<0.001	0.7116(0.6548-0.7734)	<0.001	<0.001	0.958
Total cholesterol to total lipids ratio in medium HDL	Simple mode	0.7425	0.9526(0.7134-1.2719)			
Total cholesterol to total lipids ratio in medium HDL	Weighted mode	<0.001	0.7630(0.7009-0.8306)			
Cholesterol esters in medium HDL	MR Egger	<0.001	0.6892(0.5862-0.8102)			
Cholesterol esters in medium HDL	Weighted median	<0.001	0.7436(0.6810-0.8120)			
Cholesterol esters in medium HDL	Inverse variance weighted	<0.001	0.7262(0.6620-0.7966)	<0.001	<0.001	0.4409
Cholesterol esters in medium HDL	Simple mode	0.9192	1.0111(0.8176-1.2504)			
Cholesterol esters in medium HDL	Weighted mode	<0.001	0.7736(0.6826-0.8767)			
Cholesteryl esters to total lipids ratio in medium HDL	MR Egger	<0.001	0.7557(0.6736-0.8478)			
Cholesteryl esters to total lipids ratio in medium HDL	Weighted median	<0.001	0.7742(0.7119-0.8419)			
Cholesteryl esters to total lipids ratio in medium HDL	Inverse variance weighted	<0.001	0.7252(0.6725-0.7821)	<0.001	<0.001	0.3542
Cholesteryl esters to total lipids ratio in medium HDL	Simple mode	0.258	0.8414(0.6246-1.1334)			
Cholesteryl esters to total lipids ratio in medium HDL	Weighted mode	<0.001	0.8241(0.7629-0.8903)			

Metabolites	Method	P-value	OR(95%CI)	Heterogeneity		Pleiotropy
				MR Egger Cochrane’s Q P-value	IVW Cochrane’s Q P-value	Egger intercept P-value
Free cholesterol in medium HDL	MR Egger	<0.001	0.7356(0.6304-0.8583)			
Free cholesterol in medium HDL	Weighted median	<0.001	0.8405(0.7691-0.9185)			
Free cholesterol in medium HDL	Inverse variance weighted	<0.001	0.8378(0.7696-0.9119)	<0.001	<0.001	0.0514
Free cholesterol in medium HDL	Simple mode	0.4639	0.9334(0.7765-1.1219)			
Free cholesterol in medium HDL	Weighted mode	0.0334	0.8641(0.7565-0.9870)			
Free cholesterol to total lipids ratio in medium HDL	MR Egger	0.1902	0.8451(0.6581-1.0854)			
Free cholesterol to total lipids ratio in medium HDL	Weighted median	0.1796	0.9331(0.8433-1.0324)			
Free cholesterol to total lipids ratio in medium HDL	Inverse variance weighted	0.02	0.8516(0.7439-0.9750)	<0.001	<0.001	0.9432
Free cholesterol to total lipids ratio in medium HDL	Simple mode	0.5042	1.0736(0.8723-1.3213)			
Free cholesterol to total lipids ratio in medium HDL	Weighted mode	0.9734	0.9976(0.8665-1.1485)			
Total lipids in medium HDL	MR Egger	0.0015	0.7398(0.6166-0.8877)			
Total lipids in medium HDL	Weighted median	0.0014	0.8594(0.7831-0.9433)			
Total lipids in medium HDL	Inverse variance weighted	<0.001	0.8191(0.7408-0.9057)	<0.001	<0.001	0.1922
Total lipids in medium HDL	Simple mode	0.7669	0.9728(0.8112-1.1667)			
Total lipids in medium HDL	Weighted mode	0.108	0.9017(0.7955-1.0220)			
Concentration of medium HDL particles	MR Egger	0.0085	0.7688(0.6340-0.9321)			
Concentration of medium HDL particles	Weighted median	0.0013	0.8606(0.7852-0.9432)			
Concentration of medium	Inverse variance	<0.001	0.8306(0.7486-0.9216)	<0.001	<0.001	0.3517

Metabolites	Method	P-value	OR(95%CI)	Heterogeneity		Pleiotropy
				MR Egger Cochrane’s Q P-value	IVW Cochrane’s Q P-value	Egger intercept P-value
HDL particles	weighted					
Concentration of medium HDL particles	Simple mode	0.662	0.9570(0.7862-1.1649)			
Concentration of medium HDL particles	Weighted mode	0.0891	0.8962(0.7906-1.0158)			
Phospholipids in medium HDL	MR Egger	0.006	0.7577(0.6240-0.9200)			
Phospholipids in medium HDL	Weighted median	0.0082	0.8879(0.8130-0.9697)			
Phospholipids in medium HDL	Inverse variance weighted	0.0016	0.8446(0.7607-0.9377)	<0.001	<0.001	0.1962
Phospholipids in medium HDL	Simple mode	0.5414	0.9473(0.7966-1.1265)			
Phospholipids in medium HDL	Weighted mode	0.1777	0.9278(0.8325-1.0340)			
Phospholipids to total lipids ratio in medium HDL	MR Egger	0.0072	1.2946(1.0777-1.5551)			
Phospholipids to total lipids ratio in medium HDL	Weighted median	0.0015	1.1723(1.0629-1.2928)			
Phospholipids to total lipids ratio in medium HDL	Inverse variance weighted	<0.001	1.3732(1.2278-1.5357)	<0.001	<0.001	0.4283
Phospholipids to total lipids ratio in medium HDL	Simple mode	0.9365	1.0098(0.7951-1.2826)			
Phospholipids to total lipids ratio in medium HDL	Weighted mode	0.0045	1.1304(1.0412-1.2273)			
Triglycerides in medium HDL	MR Egger	<0.001	1.2810(1.1263-1.4569)			
Triglycerides in medium HDL	Weighted median	<0.001	1.3571(1.2508-1.4725)			
Triglycerides in medium HDL	Inverse variance weighted	<0.001	1.2658(1.1674-1.3725)	<0.001	<0.001	0.8153
Triglycerides in medium HDL	Simple mode	0.0318	1.3303(1.0279-1.7217)			
Triglycerides in medium HDL	Weighted mode	<0.001	1.3891(1.2809-1.5065)			

Metabolites	Method	P-value	OR(95%CI)	Heterogeneity		Pleiotropy
				MR Egger Cochrane’s Q P-value	IVW Cochrane’s Q P-value	Egger intercept P-value
Triglycerides to total lipids ratio in medium HDL	MR Egger	<0.001	1.2995(1.1341-1.4890)			
Triglycerides to total lipids ratio in medium HDL	Weighted median	<0.001	1.2719(1.1707-1.3818)			
Triglycerides to total lipids ratio in medium HDL	Inverse variance weighted	<0.001	1.2930(1.1859-1.4097)	<0.001	<0.001	0.9259
Triglycerides to total lipids ratio in medium HDL	Simple mode	0.9357	1.0111(0.7742-1.3203)			
Triglycerides to total lipids ratio in medium HDL	Weighted mode	<0.001	1.3601(1.2664-1.4608)			
Total cholesterol in medium LDL	MR Egger	<0.001	1.5733(1.4359-1.7238)			
Total cholesterol in medium LDL	Weighted median	<0.001	1.4391(1.3343-1.5522)			
Total cholesterol in medium LDL	Inverse variance weighted	<0.001	1.4773(1.3829-1.5780)	<0.001	<0.001	0.0548
Total cholesterol in medium LDL	Simple mode	0.0421	1.2398(1.0095-1.5226)			
Total cholesterol in medium LDL	Weighted mode	<0.001	1.4482(1.3630-1.5386)			
Cholesterol esters in medium LDL	MR Egger	<0.001	1.5750(1.4394-1.7234)			
Cholesterol esters in medium LDL	Weighted median	<0.001	1.4350(1.3331-1.5448)			
Cholesterol esters in medium LDL	Inverse variance weighted	<0.001	1.4817(1.3884-1.5814)	<0.001	<0.001	0.0585
Cholesterol esters in medium LDL	Simple mode	0.0528	1.2195(0.9992-1.4883)			
Cholesterol esters in medium LDL	Weighted mode	<0.001	1.4381(1.3534-1.5280)			
Triglycerides in medium LDL	MR Egger	<0.001	1.4179(1.2503-1.6079)			
Triglycerides in medium LDL	Weighted median	<0.001	1.1697(1.0823-1.2641)			
Triglycerides in medium	Inverse variance	<0.001	1.3690(1.2589-1.4887)	<0.001	<0.001	0.4639

Metabolites	Method	P-value	OR(95%CI)	Heterogeneity		Pleiotropy
				MR Egger Cochrane's Q P-value	IVW Cochrane's Q P-value	Egger intercept P-value
LDL	weighted					
Triglycerides in medium LDL	Simple mode	0.0039	1.3074(1.0932-1.5636)			
Triglycerides in medium LDL	Weighted mode	<0.001	1.1184(1.0521-1.1889)			
Triglycerides to total lipids ratio in medium LDL	MR Egger	0.1796	0.8659(0.7033-1.0662)			
Triglycerides to total lipids ratio in medium LDL	Weighted median	0.8212	1.0089(0.9345-1.0892)			
Triglycerides to total lipids ratio in medium LDL	Inverse variance weighted	0.0338	0.8486(0.7293-0.9875)	<0.001	<0.001	0.7804
Triglycerides to total lipids ratio in medium LDL	Simple mode	0.7056	1.0536(0.8045-1.3798)			
Triglycerides to total lipids ratio in medium LDL	Weighted mode	0.4421	1.0351(0.9485-1.1296)			
Monounsaturated fatty acids (16:1, 18:1) levels	MR Egger	<0.001	1.4339(1.2147-1.6925)			
Monounsaturated fatty acids (16:1, 18:1) levels	Weighted median	<0.001	1.3797(1.2670-1.5024)			
Monounsaturated fatty acids (16:1, 18:1) levels	Inverse variance weighted	<0.001	1.3218(1.1914-1.4666)	<0.001	<0.001	0.2204
Monounsaturated fatty acids (16:1, 18:1) levels	Simple mode	0.012	1.3608(1.0739-1.7242)			
Monounsaturated fatty acids (16:1, 18:1) levels	Weighted mode	<0.001	1.4003(1.2786-1.5337)			
Total cholesterol in medium VLDL	MR Egger	<0.001	1.3378(1.1628-1.5390)			
Total cholesterol in medium VLDL	Weighted median	<0.001	1.4430(1.3376-1.5568)			
Total cholesterol in medium VLDL	Inverse variance weighted	<0.001	1.3753(1.2575-1.5041)	<0.001	<0.001	0.6152
Total cholesterol in medium VLDL	Simple mode	<0.001	1.5494(1.2644-1.8985)			
Total cholesterol in medium VLDL	Weighted mode	<0.001	1.4955(1.4010-1.5964)			

Metabolites	Method	P-value	OR(95%CI)	Heterogeneity		Pleiotropy
				MR Egger Cochrane’s Q P-value	IVW Cochrane’s Q P-value	Egger intercept P-value
Cholesterol esters in medium VLDL	MR Egger	<0.001	1.4601(1.2459-1.7113)			
Cholesterol esters in medium VLDL	Weighted median	<0.001	1.4794(1.3703-1.5971)			
Cholesterol esters in medium VLDL	Inverse variance weighted	<0.001	1.4152(1.2828-1.5613)	<0.001	<0.001	0.6235
Cholesterol esters in medium VLDL	Simple mode	<0.001	1.4321(1.1680-1.7559)			
Cholesterol esters in medium VLDL	Weighted mode	<0.001	1.5017(1.4012-1.6094)			
Free cholesterol in medium VLDL	MR Egger	0.0012	1.2588(1.0981-1.4430)			
Free cholesterol in medium VLDL	Weighted median	<0.001	1.4261(1.3222-1.5381)			
Free cholesterol in medium VLDL	Inverse variance weighted	<0.001	1.3212(1.2075-1.4456)	<0.001	<0.001	0.3572
Free cholesterol in medium VLDL	Simple mode	<0.001	1.5124(1.2480-1.8328)			
Free cholesterol in medium VLDL	Weighted mode	<0.001	1.4298(1.3400-1.5256)			
Free cholesterol to total lipids ratio in medium VLDL	MR Egger	0.0287	1.1946(1.0211-1.3976)			
Free cholesterol to total lipids ratio in medium VLDL	Weighted median	<0.001	1.1424(1.0571-1.2346)			
Free cholesterol to total lipids ratio in medium VLDL	Inverse variance weighted	<0.001	1.2513(1.1260-1.3905)	<0.001	<0.001	0.4352
Free cholesterol to total lipids ratio in medium VLDL	Simple mode	0.2921	1.1441(0.8918-1.4676)			
Free cholesterol to total lipids ratio in medium VLDL	Weighted mode	0.0023	1.1243(1.0448-1.2097)			
Total lipids in medium VLDL	MR Egger	0.0024	1.2448(1.0832-1.4304)			
Total lipids in medium VLDL	Weighted median	<0.001	1.4298(1.3237-1.5444)			
Total lipids in medium	Inverse variance	<0.001	1.3302(1.2137-1.4578)	<0.001	<0.001	0.2162

Metabolites	Method	P-value	OR(95%CI)	Heterogeneity		Pleiotropy
				MR Egger Cochrane’s Q P-value	IVW Cochrane’s Q P-value	Egger intercept P-value
VLDL	weighted					
Total lipids in medium VLDL	Simple mode	<0.001	1.4845(1.2220-1.8034)			
Total lipids in medium VLDL	Weighted mode	<0.001	1.4249(1.3262-1.5310)			
Concentration of medium VLDL particles	MR Egger	0.0023	1.2448(1.0843-1.4290)			
Concentration of medium VLDL particles	Weighted median	<0.001	1.4306(1.3287-1.5404)			
Concentration of medium VLDL particles	Inverse variance weighted	<0.001	1.3203(1.2058-1.4457)	<0.001	<0.001	0.2694
Concentration of medium VLDL particles	Simple mode	<0.001	1.4555(1.1758-1.8019)			
Concentration of medium VLDL particles	Weighted mode	<0.001	1.4352(1.3376-1.5400)			
Phospholipids in medium VLDL	MR Egger	<0.001	1.2693(1.1100-1.4514)			
Phospholipids in medium VLDL	Weighted median	<0.001	1.4283(1.3274-1.5368)			
Phospholipids in medium VLDL	Inverse variance weighted	<0.001	1.3390(1.2264-1.4619)	<0.001	<0.001	0.3026
Phospholipids in medium VLDL	Simple mode	<0.001	1.4594(1.2103-1.7598)			
Phospholipids in medium VLDL	Weighted mode	<0.001	1.4594(1.3577-1.5687)			
Triglycerides in medium VLDL	MR Egger	0.0085	1.2051(1.0508-1.3822)			
Triglycerides in medium VLDL	Weighted median	<0.001	1.4163(1.3201-1.5195)			
Triglycerides in medium VLDL	Inverse variance weighted	<0.001	1.2599(1.1527-1.3771)	<0.001	<0.001	0.4047
Triglycerides in medium VLDL	Simple mode	<0.001	1.5692(1.2641-1.9479)			
Triglycerides in medium VLDL	Weighted mode	<0.001	1.3871(1.2966-1.4840)			
Triglycerides to total lipids ratio in medium VLDL	MR Egger	<0.001	0.6563(0.5414-0.7957)			
Triglycerides to total lipids ratio in medium	Weighted median	0.0104	0.8903(0.8146-0.9731)			

Metabolites	Method	P-value	OR(95%CI)	Heterogeneity		Pleiotropy
				MR Egger Cochrane’s Q P-value	IVW Cochrane’s Q P-value	Egger intercept P-value
VLDL						
Triglycerides to total lipids ratio in medium VLDL	Inverse variance weighted	<0.001	0.7587(0.6720-0.8565)	<0.001	<0.001	0.0619
Triglycerides to total lipids ratio in medium VLDL	Simple mode	0.7466	0.9645(0.7749-1.2004)			
Triglycerides to total lipids ratio in medium VLDL	Weighted mode	0.1247	0.9415(0.8723-1.0162)			
Phosphatidylcholine and other choline levels	MR Egger	<0.001	1.2928(1.1138-1.5007)			
Phosphatidylcholine and other choline levels	Weighted median	<0.001	1.1560(1.0770-1.2408)			
Phosphatidylcholine and other choline levels	Inverse variance weighted	<0.001	1.2099(1.1066-1.3228)	<0.001	<0.001	0.2785
Phosphatidylcholine and other choline levels	Simple mode	0.0187	1.2901(1.0460-1.5911)			
Phosphatidylcholine and other choline levels	Weighted mode	0.0373	1.0844(1.0055-1.1695)			
Serum total triglyceride levels	MR Egger	<0.001	1.2881(1.1228-1.4776)			
Serum total triglyceride levels	Weighted median	<0.001	1.4273(1.3231-1.5398)			
Serum total triglyceride levels	Inverse variance weighted	<0.001	1.3310(1.2175-1.4551)	<0.001	<0.001	0.5388
Serum total triglyceride levels	Simple mode	<0.001	1.5307(1.2737-1.8395)			
Serum total triglyceride levels	Weighted mode	<0.001	1.4459(1.3572-1.5404)			
Saturated fatty acids	MR Egger	<0.001	1.4735(1.2338-1.7596)			
Saturated fatty acids	Weighted median	<0.001	1.3302(1.2107-1.4614)			
Saturated fatty acids	Inverse variance weighted	<0.001	1.4417(1.3009-1.5977)	<0.001	<0.001	0.7679
Saturated fatty acids	Simple mode	0.005	1.5711(1.1525-2.1419)			
Saturated fatty acids	Weighted mode	0.0981	1.1805(0.9712-1.4349)			
Cholesterol esters in small HDL	MR Egger	<0.001	1.8985(1.5420-2.3374)			
Cholesterol esters in small HDL	Weighted median	<0.001	1.7408(1.5905-1.9053)			

Metabolites	Method	P-value	OR(95%CI)	Heterogeneity		Pleiotropy
				MR Egger Cochrane’s Q P-value	IVW Cochrane’s Q P-value	Egger intercept P-value
Cholesterol esters in small HDL	Inverse variance weighted	<0.001	1.6243(1.4159-1.8633)	<0.001	<0.001	0.0555
Cholesterol esters in small HDL	Simple mode	0.9736	1.0050(0.7474-1.3514)			
Cholesterol esters in small HDL	Weighted mode	<0.001	1.6858(1.5217-1.8676)			
Free cholesterol in small HDL	MR Egger	0.0027	0.6724(0.5228-0.8650)			
Free cholesterol in small HDL	Weighted median	0.1292	0.9230(0.8323-1.0237)			
Free cholesterol in small HDL	Inverse variance weighted	<0.001	0.7474(0.6404-0.8723)	<0.001	<0.001	0.3003
Free cholesterol in small HDL	Simple mode	0.1996	0.8605(0.6852-1.0808)			
Free cholesterol in small HDL	Weighted mode	0.312	0.9466(0.8515-1.0523)			
Phospholipids in small HDL	MR Egger	<0.001	0.6623(0.5623-0.7800)			
Phospholipids in small HDL	Weighted median	0.0134	0.9020(0.8311-0.9788)			
Phospholipids in small HDL	Inverse variance weighted	<0.001	0.7463(0.6736-0.8268)	<0.001	<0.001	0.0709
Phospholipids in small HDL	Simple mode	0.2034	0.8790(0.7213-1.0711)			
Phospholipids in small HDL	Weighted mode	0.1939	0.9377(0.8515-1.0327)			
Triglycerides in small HDL	MR Egger	<0.001	1.2795(1.1359-1.4412)			
Triglycerides in small HDL	Weighted median	<0.001	1.3380(1.2373-1.4468)			
Triglycerides in small HDL	Inverse variance weighted	<0.001	1.3174(1.2211-1.4213)	<0.001	<0.001	0.5328
Triglycerides in small HDL	Simple mode	0.0338	1.2499(1.0192-1.5327)			
Triglycerides in small HDL	Weighted mode	<0.001	1.3528(1.2667-1.4447)			
Triglycerides to total lipids ratio in small HDL	MR Egger	<0.001	1.2825(1.1186-1.4705)			
Triglycerides to total lipids ratio in small HDL	Weighted median	<0.001	1.3349(1.2285-1.4505)			

Metabolites	Method	P-value	OR(95%CI)	Heterogeneity		Pleiotropy
				MR Egger Cochrane’s Q P-value	IVW Cochrane’s Q P-value	Egger intercept P-value
Triglycerides to total lipids ratio in small HDL	Inverse variance weighted	<0.001	1.3050(1.1986-1.4208)	<0.001	<0.001	0.7508
Triglycerides to total lipids ratio in small HDL	Simple mode	0.0642	1.2500(0.9886-1.5806)			
Triglycerides to total lipids ratio in small HDL	Weighted mode	<0.001	1.3574(1.2606-1.4615)			
Triglycerides in small LDL	MR Egger	<0.001	1.4145(1.2373-1.6171)			
Triglycerides in small LDL	Weighted median	<0.001	1.2212(1.1353-1.3136)			
Triglycerides in small LDL	Inverse variance weighted	<0.001	1.3425(1.2309-1.4641)	<0.001	<0.001	0.3164
Triglycerides in small LDL	Simple mode	0.0269	1.2763(1.0305-1.5809)			
Triglycerides in small LDL	Weighted mode	<0.001	1.1538(1.0663-1.2484)			
Total cholesterol in small VLDL	MR Egger	<0.001	1.6672(1.4928-1.8620)			
Total cholesterol in small VLDL	Weighted median	<0.001	1.5216(1.4119-1.6398)			
Total cholesterol in small VLDL	Inverse variance weighted	<0.001	1.5393(1.4383-1.6474)	<0.001	<0.001	0.0755
Total cholesterol in small VLDL	Simple mode	0.004	1.3978(1.1161-1.7507)			
Total cholesterol in small VLDL	Weighted mode	<0.001	1.5492(1.4276-1.6812)			
Free cholesterol in small VLDL	MR Egger	<0.001	1.4671(1.3162-1.6353)			
Free cholesterol in small VLDL	Weighted median	<0.001	1.4556(1.3477-1.5721)			
Free cholesterol in small VLDL	Inverse variance weighted	<0.001	1.4112(1.3179-1.5110)	<0.001	<0.001	0.3671
Free cholesterol in small VLDL	Simple mode	<0.001	1.4656(1.2196-1.7611)			
Free cholesterol in small VLDL	Weighted mode	<0.001	1.4799(1.3822-1.5845)			
Total lipids in small VLDL	MR Egger	<0.001	1.4197(1.2468-1.6165)			
Total lipids in small VLDL	Weighted median	<0.001	1.4475(1.3457-1.5570)			

Metabolites	Method	P-value	OR(95%CI)	Heterogeneity		Pleiotropy
				MR Egger Cochrane’s Q P-value	IVW Cochrane’s Q P-value	Egger intercept P-value
Total lipids in small VLDL	Inverse variance weighted	<0.001	1.4075(1.2949-1.5300)	<0.001	<0.001	0.8652
Total lipids in small VLDL	Simple mode	<0.001	1.4940(1.2726-1.7539)			
Total lipids in small VLDL	Weighted mode	<0.001	1.4940(1.4024-1.5916)			
Concentration of small VLDL particles	MR Egger	<0.001	1.3988(1.2290-1.5921)			
Concentration of small VLDL particles	Weighted median	<0.001	1.4368(1.3364-1.5447)			
Concentration of small VLDL particles	Inverse variance weighted	<0.001	1.3789(1.2679-1.4996)	<0.001	<0.001	0.7752
Concentration of small VLDL particles	Simple mode	<0.001	1.5640(1.2937-1.8907)			
Concentration of small VLDL particles	Weighted mode	<0.001	1.4793(1.3758-1.5905)			
Phospholipids in small VLDL	MR Egger	<0.001	1.3889(1.2285-1.5704)			
Phospholipids in small VLDL	Weighted median	<0.001	1.4343(1.3350-1.5410)			
Phospholipids in small VLDL	Inverse variance weighted	<0.001	1.3676(1.2655-1.4779)	<0.001	<0.001	0.7491
Phospholipids in small VLDL	Simple mode	<0.001	1.4901(1.2078-1.8384)			
Phospholipids in small VLDL	Weighted mode	<0.001	1.4527(1.3586-1.5534)			
Phospholipids to total lipids ratio in small VLDL	MR Egger	<0.001	0.5951(0.4745-0.7465)			
Phospholipids to total lipids ratio in small VLDL	Weighted median	<0.001	0.7223(0.6538-0.7979)			
Phospholipids to total lipids ratio in small VLDL	Inverse variance weighted	<0.001	0.6493(0.5722-0.7369)	<0.001	<0.001	0.365
Phospholipids to total lipids ratio in small VLDL	Simple mode	0.6888	0.9460(0.7213-1.2407)			
Phospholipids to total lipids ratio in small	Weighted mode	0.2759	0.8932(0.7297-1.0934)			

Metabolites	Method	P-value	OR(95%CI)	Heterogeneity		Pleiotropy
				MR Egger Cochrane’s Q P-value	IVW Cochrane’s Q P-value	Egger intercept P-value
VLDL						
Triglycerides in small VLDL	MR Egger	<0.001	1.2716(1.1162-1.4487)			
Triglycerides in small VLDL	Weighted median	<0.001	1.4031(1.3098-1.5030)			
Triglycerides in small VLDL	Inverse variance weighted	<0.001	1.2886(1.1823-1.4044)	<0.001	<0.001	0.7908
Triglycerides in small VLDL	Simple mode	<0.001	1.4727(1.1842-1.8315)			
Triglycerides in small VLDL	Weighted mode	<0.001	1.4281(1.3349-1.5279)			
Ratio of triglycerides to phosphoglycerides	MR Egger	0.0032	1.2220(1.0715-1.3937)			
Ratio of triglycerides to phosphoglycerides	Weighted median	<0.001	1.3936(1.2967-1.4977)			
Ratio of triglycerides to phosphoglycerides	Inverse variance weighted	<0.001	1.2297(1.1317-1.3361)	<0.001	<0.001	0.9045
Ratio of triglycerides to phosphoglycerides	Simple mode	0.0947	1.2231(0.9672-1.5467)			
Ratio of triglycerides to phosphoglycerides	Weighted mode	<0.001	1.4118(1.3039-1.5286)			
Total cholines levels	MR Egger	<0.001	1.3787(1.1985-1.5861)			
Total cholines levels	Weighted median	<0.001	1.1651(1.0785-1.2585)			
Total cholines levels	Inverse variance weighted	<0.001	1.2708(1.1686-1.3819)	<0.001	<0.001	0.1574
Total cholines levels	Simple mode	0.8733	0.9814(0.7793-1.2358)			
Total cholines levels	Weighted mode	0.092	1.0763(0.9886-1.1716)			
Total fatty acids	MR Egger	<0.001	1.5454(1.3316-1.7934)			
Total fatty acids	Weighted median	<0.001	1.3340(1.2266-1.4509)			
Total fatty acids	Inverse variance weighted	<0.001	1.3986(1.2794-1.5288)	<0.001	<0.001	0.1044
Total fatty acids	Simple mode	0.0186	1.3711(1.0574-1.7779)			
Total fatty acids	Weighted mode	<0.001	1.4958(1.3053-1.7142)			
Total phosphoglycerides levels	MR Egger	0.0017	1.3044(1.1092-1.5339)			
Total phosphoglycerides levels	Weighted median	<0.001	1.1604(1.0757-1.2519)			
Total phosphoglycerides levels	Inverse variance weighted	<0.001	1.2515(1.1371-1.3773)	<0.001	<0.001	0.5349
Total phosphoglycerides	Simple mode	0.4286	1.0844(0.8878-1.3245)			

Metabolites	Method	P-value	OR(95%CI)	Heterogeneity		Pleiotropy
				MR Egger Cochrane’s Q P-value	IVW Cochrane’s Q P-value	Egger intercept P-value
levels						
Total phosphoglycerides levels	Weighted mode	0.0497	1.0750(1.0008-1.1547)			
Total cholesterol levels in VLDL	MR Egger	<0.001	1.5687(1.3532-1.8186)			
Total cholesterol levels in VLDL	Weighted median	<0.001	1.5264(1.4174-1.6438)			
Total cholesterol levels in VLDL	Inverse variance weighted	<0.001	1.4944(1.3657-1.6353)	<0.001	<0.001	0.418
Total cholesterol levels in VLDL	Simple mode	0.0019	1.3898(1.1338-1.7035)			
Total cholesterol levels in VLDL	Weighted mode	<0.001	1.5225(1.4165-1.6363)			
Triglyceride levels in VLDL	MR Egger	0.0038	1.2186(1.0684-1.3899)			
Triglyceride levels in VLDL	Weighted median	<0.001	1.4276(1.3310-1.5312)			
Triglyceride levels in VLDL	Inverse variance weighted	<0.001	1.2850(1.1783-1.4014)	<0.001	<0.001	0.2944
Triglyceride levels in VLDL	Simple mode	<0.001	1.5900(1.3090-1.9314)			
Triglyceride levels in VLDL	Weighted mode	<0.001	1.4384(1.3432-1.5403)			
Cholesteryl esters to total lipids ratio in very large HDL	MR Egger	0.4196	1.0705(0.9078-1.2625)			
Cholesteryl esters to total lipids ratio in very large HDL	Weighted median	0.5065	1.0290(0.9457-1.1198)			
Cholesteryl esters to total lipids ratio in very large HDL	Inverse variance weighted	<0.001	1.1977(1.0831-1.3244)	<0.001	<0.001	0.0963
Cholesteryl esters to total lipids ratio in very large HDL	Simple mode	0.6909	0.9528(0.7513-1.2085)			
Cholesteryl esters to total lipids ratio in very large HDL	Weighted mode	0.2041	0.9405(0.8559-1.0334)			
Total lipids in very large HDL	MR Egger	0.7427	0.9819(0.8806-1.0948)			

Metabolites	Method	P-value	OR(95%CI)	Heterogeneity		Pleiotropy
				MR Egger Cochrane' s Q P-value	IVW Cochrane' s Q P-value	Egger intercept P-value
Total lipids in very large HDL	Weighted median	0.7504	0.9883(0.9194-1.0625)			
Total lipids in very large HDL	Inverse variance weighted	0.0319	0.9262(0.8636-0.9934)	<0.001	<0.001	0.1724
Total lipids in very large HDL	Simple mode	0.6885	0.9574(0.7741-1.1841)			
Total lipids in very large HDL	Weighted mode	0.648	1.0135(0.9569-1.0734)			
Concentration of very large HDL particles	MR Egger	0.7909	0.9853(0.8836-1.0988)			
Concentration of very large HDL particles	Weighted median	0.7282	0.9884(0.9257-1.0554)			
Concentration of very large HDL particles	Inverse variance weighted	0.0308	0.9252(0.8621-0.9928)	<0.001	<0.001	0.1393
Concentration of very large HDL particles	Simple mode	0.6673	0.9554(0.7761-1.1761)			
Concentration of very large HDL particles	Weighted mode	0.749	1.0100(0.9502-1.0737)			
Phospholipids in very large HDL	MR Egger	0.0524	0.9044(0.8177-1.0003)			
Phospholipids in very large HDL	Weighted median	0.1895	0.9538(0.8886-1.0237)			
Phospholipids in very large HDL	Inverse variance weighted	<0.001	0.8662(0.8128-0.9232)	<0.001	<0.001	0.281
Phospholipids in very large HDL	Simple mode	0.1526	0.8487(0.6785-1.0616)			
Phospholipids in very large HDL	Weighted mode	0.2212	0.9669(0.9162-1.0203)			
Phospholipids to total lipids ratio in very large HDL	MR Egger	0.0627	0.8267(0.6778-1.0083)			
Phospholipids to total lipids ratio in very large HDL	Weighted median	<0.001	0.7442(0.6712-0.8252)			
Phospholipids to total lipids ratio in very large HDL	Inverse variance weighted	<0.001	0.7536(0.6726-0.8442)	<0.001	<0.001	0.2676
Phospholipids to total lipids ratio in very large HDL	Simple mode	0.0744	0.7205(0.5042-1.0296)			

Metabolites	Method	P-value	OR(95%CI)	Heterogeneity		Pleiotropy
				MR Egger Cochrane’s Q P-value	IVW Cochrane’s Q P-value	Egger intercept P-value
Phospholipids to total lipids ratio in very large HDL	Weighted mode	0.7619	0.9436(0.6486-1.3727)			
Triglycerides in very large HDL	MR Egger	0.1814	1.1282(0.9464-1.3450)			
Triglycerides in very large HDL	Weighted median	0.0049	1.1016(1.0297-1.1785)			
Triglycerides in very large HDL	Inverse variance weighted	0.0112	1.1629(1.0349-1.3067)	<0.001	<0.001	0.6518
Triglycerides in very large HDL	Simple mode	0.3407	0.9098(0.7496-1.1041)			
Triglycerides in very large HDL	Weighted mode	0.012	1.0747(1.0170-1.1357)			
Triglycerides to total lipids ratio in very large HDL	MR Egger	0.0025	1.3916(1.1285-1.7161)			
Triglycerides to total lipids ratio in very large HDL	Weighted median	<0.001	1.4503(1.3246-1.5879)			
Triglycerides to total lipids ratio in very large HDL	Inverse variance weighted	<0.001	1.3339(1.1844-1.5022)	<0.001	<0.001	0.6309
Triglycerides to total lipids ratio in very large HDL	Simple mode	0.532	1.0967(0.8218-1.4635)			
Triglycerides to total lipids ratio in very large HDL	Weighted mode	<0.001	1.5623(1.4126-1.7277)			
Total cholesterol in very large VLDL	MR Egger	0.093	1.1557(0.9774-1.3665)			
Total cholesterol in very large VLDL	Weighted median	<0.001	1.5113(1.3979-1.6338)			
Total cholesterol in very large VLDL	Inverse variance weighted	<0.001	1.2786(1.1489-1.4229)	<0.001	<0.001	0.1287
Total cholesterol in very large VLDL	Simple mode	<0.001	1.5965(1.2813-1.9893)			
Total cholesterol in very large VLDL	Weighted mode	<0.001	1.4977(1.3813-1.6239)			
Cholesterol esters in very large VLDL	MR Egger	0.0296	1.2065(1.0208-1.4260)			

Metabolites	Method	P-value	OR(95%CI)	Heterogeneity		Pleiotropy
				MR Egger Cochrane’s Q P-value	IVW Cochrane’s Q P-value	Egger intercept P-value
Cholesterol esters in very large VLDL	Weighted median	<0.001	1.5050(1.3927-1.6263)			
Cholesterol esters in very large VLDL	Inverse variance weighted	<0.001	1.2560(1.1300-1.3961)	<0.001	<0.001	0.5429
Cholesterol esters in very large VLDL	Simple mode	<0.001	1.6054(1.2727-2.0251)			
Cholesterol esters in very large VLDL	Weighted mode	<0.001	1.5337(1.4208-1.6556)			
Free cholesterol in very large VLDL	MR Egger	0.1459	1.1391(0.9568-1.3562)			
Free cholesterol in very large VLDL	Weighted median	<0.001	1.4760(1.3607-1.6010)			
Free cholesterol in very large VLDL	Inverse variance weighted	<0.001	1.3005(1.1637-1.4534)	<0.001	<0.001	0.0575
Free cholesterol in very large VLDL	Simple mode	<0.001	1.5491(1.2297-1.9515)			
Free cholesterol in very large VLDL	Weighted mode	<0.001	1.5128(1.3901-1.6464)			
Free cholesterol to total lipids ratio in very large VLDL	MR Egger	0.0243	1.7119(1.0839-2.7039)			
Free cholesterol to total lipids ratio in very large VLDL	Weighted median	0.4796	1.0542(0.9107-1.2202)			
Free cholesterol to total lipids ratio in very large VLDL	Inverse variance weighted	0.0019	1.3846(1.1273-1.7006)	<0.001	<0.001	0.3121
Free cholesterol to total lipids ratio in very large VLDL	Simple mode	0.592	0.9216(0.6849-1.2402)			
Free cholesterol to total lipids ratio in very large VLDL	Weighted mode	0.9877	1.0015(0.8287-1.2104)			
Total lipids in very large VLDL	MR Egger	0.1387	1.1350(0.9609-1.3406)			
Total lipids in very large VLDL	Weighted median	<0.001	1.4700(1.3601-1.5888)			
Total lipids in very large VLDL	Inverse variance weighted	<0.001	1.2605(1.1324-1.4030)	<0.001	<0.001	0.111
Total lipids in very	Simple mode	<0.001	1.4831(1.1933-1.8432)			

Metabolites	Method	P-value	OR(95%CI)	Heterogeneity		Pleiotropy
				MR Egger Cochrane's Q P-value	IVW Cochrane's Q P-value	Egger intercept P-value
large VLDL						
Total lipids in very large VLDL	Weighted mode	<0.001	1.4690(1.3631-1.5831)			
Concentration of very large VLDL particles	MR Egger	0.1472	1.1318(0.9584-1.3365)			
Concentration of very large VLDL particles	Weighted median	<0.001	1.4780(1.3634-1.6022)			
Concentration of very large VLDL particles	Inverse variance weighted	<0.001	1.2644(1.1358-1.4076)	<0.001	<0.001	0.0917
Concentration of very large VLDL particles	Simple mode	<0.001	1.5362(1.2222-1.9309)			
Concentration of very large VLDL particles	Weighted mode	<0.001	1.4633(1.3492-1.5871)			
Phospholipids in very large VLDL	MR Egger	0.1252	1.1416(0.9649-1.3506)			
Phospholipids in very large VLDL	Weighted median	<0.001	1.4991(1.3832-1.6247)			
Phospholipids in very large VLDL	Inverse variance weighted	<0.001	1.2857(1.1546-1.4318)	<0.001	<0.001	0.0755
Phospholipids in very large VLDL	Simple mode	<0.001	1.5114(1.1965-1.9091)			
Phospholipids in very large VLDL	Weighted mode	<0.001	1.4830(1.3597-1.6175)			
Triglycerides in very large VLDL	MR Egger	0.125	1.1372(0.9660-1.3386)			
Triglycerides in very large VLDL	Weighted median	<0.001	1.4303(1.3181-1.5521)			
Triglycerides in very large VLDL	Inverse variance weighted	<0.001	1.2539(1.1293-1.3922)	<0.001	<0.001	0.1298
Triglycerides in very large VLDL	Simple mode	0.0014	1.4388(1.1574-1.7885)			
Triglycerides in very large VLDL	Weighted mode	<0.001	1.4532(1.3429-1.5726)			
Triglycerides to total lipids ratio in very large VLDL	MR Egger	0.0011	0.5424(0.3814-0.7714)			
Triglycerides to total lipids ratio in very large VLDL	Weighted median	0.0069	0.8388(0.7384-0.9529)			
Triglycerides to total	Inverse variance	<0.001	0.6948(0.5759-0.8383)	<0.001	<0.001	0.1092

Metabolites	Method	P-value	OR(95%CI)	Heterogeneity		Pleiotropy
				MR Egger Cochrane’s Q P-value	IVW Cochrane’s Q P-value	Egger intercept P-value
lipids ratio in very large VLDL	weighted					
Triglycerides to total lipids ratio in very large VLDL	Simple mode	0.2529	0.8070(0.5604-1.1622)			
Triglycerides to total lipids ratio in very large VLDL	Weighted mode	0.1018	0.8793(0.7552-1.0238)			
Total cholesterol to total lipids ratio in very small VLDL	MR Egger	0.0656	0.8144(0.6563-1.0106)			
Total cholesterol to total lipids ratio in very small VLDL	Weighted median	<0.001	0.8218(0.7439-0.9079)			
Total cholesterol to total lipids ratio in very small VLDL	Inverse variance weighted	0.0193	0.8619(0.7611-0.9762)	<0.001	<0.001	0.5294
Total cholesterol to total lipids ratio in very small VLDL	Simple mode	0.1754	0.8551(0.6831-1.0705)			
Total cholesterol to total lipids ratio in very small VLDL	Weighted mode	0.0056	0.8360(0.7386-0.9462)			
Triglycerides in very small VLDL	MR Egger	<0.001	1.3908(1.2339-1.5678)			
Triglycerides in very small VLDL	Weighted median	<0.001	1.3722(1.2734-1.4788)			
Triglycerides in very small VLDL	Inverse variance weighted	<0.001	1.3066(1.2091-1.4119)	<0.001	<0.001	0.1821
Triglycerides in very small VLDL	Simple mode	0.0084	1.4085(1.0952-1.8116)			
Triglycerides in very small VLDL	Weighted mode	<0.001	1.3731(1.2721-1.4822)			
Total cholesterol levels in chylomicrons and extremely large VLDL	MR Egger	0.0359	1.2256(1.0157-1.4790)			
Total cholesterol levels in chylomicrons and extremely large VLDL	Weighted median	<0.001	1.5339(1.4057-1.6738)			
Total cholesterol levels	Inverse variance	<0.001	1.3200(1.1748-1.4831)	<0.001	<0.001	0.3259

Metabolites	Method	P-value	OR(95%CI)	Heterogeneity		Pleiotropy
				MR Egger Cochrane’s Q P-value	IVW Cochrane’s Q P-value	Egger intercept P-value
in chylomicrons and extremely large VLDL	weighted					
Total cholesterol levels in chylomicrons and extremely large VLDL	Simple mode	0.0011	1.5135(1.1861-1.9314)			
Total cholesterol levels in chylomicrons and extremely large VLDL	Weighted mode	<0.001	1.5548(1.4368-1.6826)			
Total cholesterol to total lipids ratio in chylomicrons and extremely large VLDL	MR Egger	0.1818	1.2093(0.9174-1.5940)			
Total cholesterol to total lipids ratio in chylomicrons and extremely large VLDL	Weighted median	<0.001	1.1786(1.0787-1.2877)			
Total cholesterol to total lipids ratio in chylomicrons and extremely large VLDL	Inverse variance weighted	<0.001	1.3553(1.1478-1.6004)	<0.001	<0.001	0.3145
Total cholesterol to total lipids ratio in chylomicrons and extremely large VLDL	Simple mode	0.8667	1.0169(0.8365-1.2363)			
Total cholesterol to total lipids ratio in chylomicrons and extremely large VLDL	Weighted mode	0.0023	1.1168(1.0429-1.1959)			
Cholesteryl ester levels in chylomicrons and extremely large VLDL	MR Egger	0.007	1.3200(1.0827-1.6093)			
Cholesteryl ester levels in chylomicrons and extremely large VLDL	Weighted median	<0.001	1.5454(1.4098-1.6940)			
Cholesteryl ester levels in chylomicrons and extremely large VLDL	Inverse variance weighted	<0.001	1.3668(1.2133-1.5396)	<0.001	<0.001	0.6667
Cholesteryl ester levels in chylomicrons and extremely large VLDL	Simple mode	<0.001	1.5520(1.2167-1.9797)			

Metabolites	Method	P-value	OR(95%CI)	Heterogeneity		Pleiotropy
				MR Egger Cochrane' s Q P-value	IVW Cochrane' s Q P-value	Egger intercept P-value
Cholesteryl ester levels in chylomicrons and extremely large VLDL	Weighted mode	<0.001	1.6072(1.4831-1.7418)			
Cholesteryl esters to total lipids ratio in chylomicrons and extremely large VLDL	MR Egger	<0.001	1.6274(1.2394-2.1369)			
Cholesteryl esters to total lipids ratio in chylomicrons and extremely large VLDL	Weighted median	0.1323	1.0801(0.9770-1.1942)			
Cholesteryl esters to total lipids ratio in chylomicrons and extremely large VLDL	Inverse variance weighted	<0.001	1.4185(1.2147-1.6565)	<0.001	<0.001	0.2334
Cholesteryl esters to total lipids ratio in chylomicrons and extremely large VLDL	Simple mode	0.7456	1.0370(0.8334-1.2902)			
Cholesteryl esters to total lipids ratio in chylomicrons and extremely large VLDL	Weighted mode	0.1486	1.0688(0.9774-1.1687)			
Free cholesterol levels in chylomicrons and extremely large VLDL	MR Egger	0.173	1.1262(0.9502-1.3349)			
Free cholesterol levels in chylomicrons and extremely large VLDL	Weighted median	<0.001	1.4787(1.3601-1.6078)			
Free cholesterol levels in chylomicrons and extremely large VLDL	Inverse variance weighted	<0.001	1.2685(1.1380-1.4140)	<0.001	<0.001	0.0787
Free cholesterol levels in chylomicrons and extremely large VLDL	Simple mode	0.0191	1.3840(1.0584-1.8098)			
Free cholesterol levels in chylomicrons and extremely large VLDL	Weighted mode	<0.001	1.5065(1.3718-1.6545)			
Total lipid levels in chylomicrons and	MR Egger	0.1553	1.1468(0.9506-1.3835)			

Metabolites	Method	P-value	OR(95%CI)	Heterogeneity		Pleiotropy
				MR Egger Cochrane’s Q P-value	IVW Cochrane’s Q P-value	Egger intercept P-value
extremely large VLDL						
Total lipid levels in chylomicrons and extremely large VLDL	Weighted median	<0.001	1.4887(1.3690-1.6188)			
Total lipid levels in chylomicrons and extremely large VLDL	Inverse variance weighted	<0.001	1.2651(1.1239-1.4241)	<0.001	<0.001	0.1898
Total lipid levels in chylomicrons and extremely large VLDL	Simple mode	0.0093	1.4185(1.0948-1.8377)			
Total lipid levels in chylomicrons and extremely large VLDL	Weighted mode	<0.001	1.5069(1.3825-1.6425)			
Concentration of chylomicrons and extremely large VLDL particles	MR Egger	0.1314	1.1553(0.9590-1.3918)			
Concentration of chylomicrons and extremely large VLDL particles	Weighted median	<0.001	1.4767(1.3562-1.6080)			
Concentration of chylomicrons and extremely large VLDL particles	Inverse variance weighted	<0.001	1.2782(1.1370-1.4368)	<0.001	<0.001	0.1752
Concentration of chylomicrons and extremely large VLDL particles	Simple mode	0.0123	1.4034(1.0806-1.8226)			
Concentration of chylomicrons and extremely large VLDL particles	Weighted mode	<0.001	1.5270(1.3894-1.6783)			
Phospholipid levels in chylomicrons and extremely large VLDL	MR Egger	0.1619	1.1390(0.9502-1.3654)			
Phospholipid levels in chylomicrons and extremely large VLDL	Weighted median	<0.001	1.4918(1.3764-1.6168)			
Phospholipid levels in	Inverse variance	<0.001	1.2506(1.1147-1.4031)	<0.001	<0.001	0.1944

Metabolites	Method	P-value	OR(95%CI)	Heterogeneity		Pleiotropy
				MR Egger Cochrane’s Q P-value	IVW Cochrane’s Q P-value	Egger intercept P-value
chylomicrons and extremely large VLDL	weighted					
Phospholipid levels in chylomicrons and extremely large VLDL	Simple mode	0.001	1.5093(1.1874-1.9185)			
Phospholipid levels in chylomicrons and extremely large VLDL	Weighted mode	<0.001	1.4896(1.3676-1.6226)			
Triglyceride levels in chylomicrons and extremely large VLDL	MR Egger	0.2018	1.1369(0.9347-1.3829)			
Triglyceride levels in chylomicrons and extremely large VLDL	Weighted median	<0.001	1.4880(1.3584-1.6300)			
Triglyceride levels in chylomicrons and extremely large VLDL	Inverse variance weighted	<0.001	1.2746(1.1276-1.4408)	<0.001	<0.001	0.1466
Triglyceride levels in chylomicrons and extremely large VLDL	Simple mode	0.0153	1.4300(1.0757-1.9011)			
Triglyceride levels in chylomicrons and extremely large VLDL	Weighted mode	<0.001	1.5374(1.3962-1.6929)			
Triglycerides to total lipids ratio in chylomicrons and extremely large VLDL	MR Egger	0.0421	0.7862(0.6260-0.9873)			
Triglycerides to total lipids ratio in chylomicrons and extremely large VLDL	Weighted median	<0.001	0.8353(0.7568-0.9219)			
Triglycerides to total lipids ratio in chylomicrons and extremely large VLDL	Inverse variance weighted	0.0037	0.8165(0.7122-0.9362)	<0.001	<0.001	0.6836
Triglycerides to total lipids ratio in chylomicrons and extremely large VLDL	Simple mode	0.3978	1.0877(0.8961-1.3202)			
Triglycerides to total	Weighted mode	0.0624	0.9292(0.8612-1.0026)			

Metabolites	Method	P-value	OR(95%CI)	Heterogeneity		Pleiotropy
				MR Egger Cochrane' s Q P-value	IVW Cochrane' s Q P-value	Egger intercept P-value
lipids ratio in chylomicrons and extremely large VLDL						
